# Inhibition of SMAD3 effectively reduces ADAMTS-5 expression in the early stages of osteoarthritis

**DOI:** 10.1186/s12891-022-05949-8

**Published:** 2023-02-17

**Authors:** Wei Xiang, Chao Wang, Zhoujun Zhu, Dui Wang, Zhenyu Qiu, Weishan Wang

**Affiliations:** 1 Department of Orthopedics Center, The First Affiliated Hospital, Shihezi University School of Medicine, 107 North Second Road, Shihezi, Xinjiang, 832000 People’s Republic of China; 2 Renmin Hospital of Zhijiang, Yichang, Hubei China; 3 Shihezi University School of Medicine, Xinjiang, China; 4 Department of Joint Surgery, The Sixth Affiliated Hospital of Xinjiang Medical University, Urumqi, Xinjiang Uygur Autonomous Region China

**Keywords:** Osteoarthritis, SMAD3, ADAMTS-5, miRNA-140

## Abstract

**Objective:**

As one of the most important protein-degrading enzymes, ADAMTS-5 plays an important role in the regulation of cartilage homeostasis, while miRNA-140 is specifically expressed in cartilage, which can inhibit the expression of ADAMTS-5 and delay the progression of OA (osteoarthritis). SMAD3 is a key protein in the TGF-β signaling pathway, inhibiting the expression of miRNA-140 at the transcriptional and post-transcriptional levels, and studies have confirmed the high expression of SMAD3 in knee cartilage degeneration, but whether SMAD3 can mediate the expression of miRNA-140 to regulate ADAMTS-5 remains unknown.

**Methods:**

Sprague–Dawley (SD) rat chondrocytes were extracted in vitro and treated with a SMAD3 inhibitor (SIS3) and miRNA-140 mimics after IL-1 induction. The expression of ADAMTS-5 was detected at the protein and gene levels at 24 h, 48 h, and 72 h after treatment. The OA model of SD rats was created using the traditional Hulth method in vivo, with SIS3 and lentivirus packaged miRNA-140 mimics injected intra-articularly at 2 weeks, 6 weeks and 12 weeks after surgery. The expression of miRNA-140 and ADAMTS-5 in the knee cartilage tissue was observed at the protein and gene levels. Concurrently, knee joint specimens were fixed, decalcified, and embedded in paraffin prior to immunohistochemical, Safranin O/Fast Green staining, and HE staining analyses for ADAMTS-5 and SMAD3.

**Results:**

In vitro, the expression of ADAMTS-5 protein and mRNA in the SIS3 group decreased to different degrees at each time point. Meanwhile, the expression of miRNA-140 in the SIS3 group was significantly increased, and the expression of ADAMTS-5 in the miRNA-140 mimics group was also significantly downregulated (*P < 0.05*). In vivo, it was found that ADAMTS-5 protein and gene were downregulated to varying degrees in the SIS3 and miRNA-140 mimic groups at three time points, with the most significant decrease at the early stage (2 weeks) (*P < 0.05*), and the expression of miRNA-140 in the SIS3 group was significantly upregulated, similar to the changes detected in vitro. Immunohistochemical results showed that the expression of ADAMTS-5 protein in the SIS3 and miRNA-140 groups was significantly downregulated compared to that in the blank group. The results of hematoxylin and eosin staining showed that in the early stage, there was no obvious change in cartilage structure in the SIS3 and miRNA-140 mock groups. The same was observed in the results of Safranin O/Fast Green staining; the number of chondrocytes was not significantly reduced, and the tide line was complete.

**Conclusion:**

The results of in vitro and in vivo experiments preliminarily showed that the inhibition of SMAD3 significantly reduced the expression of ADAMTS-5 in early OA cartilage, and this regulation might be accomplished indirectly through miRNA-140.

**Supplementary Information:**

The online version contains supplementary material available at 10.1186/s12891-022-05949-8.

## Introduction

OA (osteoarthritis) is the leading cause of disability in the elderly over the age of 65 [1]. The global age-standardized incidence rate (ASIR) of OA (compared to low back and neck pain) increased by 0.32% per year (95% CI 0.28 to 0.36), or approximately 9%, and it is worth noting that the aging of the global population has actually driven a greater increase in the absolute number of new cases of OA without standardizing on age [2]. OA is a degenerative joint lesion caused by many factors, and its incidence is increasing year by year [3], but the specific pathogenesis has always been a difficult point in research. Knee OA is the most common form of osteoarthritis [4], and its most obvious pathological features are the progressive destruction of the joint cartilage structure, accompanied by osteophyte formation around the joint and varying degrees of synovitis [5, 6]. Chondrocytes are the only cell type in cartilage, and under the combined action of various factors, substructural changes in chondrocytes and subchondral bone occur, the most important of which are protein-degrading enzymes [7]. The theory of protein-degrading enzymes suggests that OA is the degradation of the extra matrix and basement membrane components of chondrocytes and subchondral bone cells rather than synthesis, and ADAMTS-5 plays a central role in the degradation of aggregate proteoglycans [8, 9]. Aggrecanase is the main proteoglycan-degrading enzyme, and inhibition of ADAMTS-4 and ADAMTS-5 can effectively prevent the degradation of OA cartilage aggrecan and prevent degeneration of cartilage [10, 11]. Studies have shown that injecting small interfering RNA (siRNA) with ADAMTS-5 through intra-articular injection can effectively delay the progression of osteoarthritis in mice [12].

Mechanical stress promotes the progression of osteoarthritis, with abnormal mechanical stress activating interleukin 1β, tumor necrosis factor α, nuclear factor-κB, Wnt, transforming growth factor β, microRNA pathways, and oxidative stress pathways that induce pathological progression in osteoarthritis [13]. Data suggest that highly active TGFβ1 activates the expression of cartilage-degrading enzymes, and abnormally activated TGFβ1 induces the formation of subchondral bone and the expansion of calcified cartilage regions, ultimately leading to degradation of cartilage tissue [14–16]. Articular cartilage and subchondral bone are functional units [17], and a growing body of research confirms that transforming growth factor β (TGFβ) plays a key regulatory role in maintaining the balance of the internal environment of articular cartilage and subchondral bone. The activation of latent TGFβ in the extracellular matrix (ECM) under pathological conditions is a prerequisite for its function [18]. Studies have found that in healthy joints and osteoarthritis joints, the concentration of active TGFβ varies greatly; the concentration of active TGFβ in healthy joints is low, while the concentration of active TGFβ in osteoarthritis joints is higher, resulting in the activation of different signaling pathways in joint cells, and high levels of TGFβ will destroy the homeostasis of cartilage and impair the metabolic activity of chondrocytes [19, 20]. Characteristic pathologies of osteoarthritis joints, such as cartilage injury, osteophyte formation, and synovial fibrosis, appear to be caused even by high levels of active TGFβ and altered signaling pathways in chondrocytes [21, 22], while Smad3 is a key protein in the TGFβ pathway, and an assessment of the cogenetic etiology between osteoarthritis and bone density suggests that SMAD3 is a new osteoarthritis risk site [23, 24]. MicroRNAs (miRNAs) are a class of small, noncoding single-stranded RNAs that, by binding to target messenger RNAs (mRNAs), exert biological effects that lead to decay or translational inhibition of target messenger RNAs [25, 26]. miR-140 has been reported in many studies to play an important role in the pathogenesis of OA [27], and the regulatory role of miRNA-140 on chondrosity homeostasis has been established, which is specifically expressed in cartilage. It has been reported in the literature that expression of miRNA-140 can promote the expression of type II collagen and inhibit the expression of ADAMTS-5 [28], thereby delaying the progression of OA to some extent.

The regulatory relationship between SMAD3 and miRNA-140 has been reported, SMAD3 can inhibits the expression of miRNA-140 at the transcriptional and posttranscriptional levels [29], while the regulatory relationship between SMAD3 and ADAMTS-5 in OA has not been reported. Whether direct regulation or indirect regulation occurs between the two is unknown, and the role of miRNA-140 in it is unknown. In this study, we mainly explored the regulatory relationship between SMAD3 and ADAMTS-5 at both the in vitro and in vivo levels. We hypothesize that SMAD3 can regulate the expression of ADAMTS-5, and this regulation may be done indirectly through miRNA-140.

## Materials and methods

### Animals

SD rats were purchased from the Animal Experimental Center. All experimental methods and procedures were approved by the Animal Experimental Ethics Inspection of First Affiliated Hospital, Shihezi University School of Medicine (A2017-130-01). SD rats were raised in a temperature-controlled dry environment (25 °C) and provided adequate food and water.

### Cell samples

Articular cartilage was extracted from the knee joints of the rats at 2 weeks of age. The tissue sample was digested with 0.2% type II collagenase (Gibco, USA) for 1 h. After centrifugation at 1000 g × 15 min, the supernatant was collected and digested with 0.25% trypsin (APExBIO, USA) overnight (≤ 12 h). Centrifugation at 1000×g for 15 min was performed to remove the supernatant. The pellet containing chondrocytes was resuspended in DMEM (Gibco, USA) to a final volume of 6 mL, and 1 mL was aliquoted into each Petri dish. The rat chondrocytes were cultured in an incubator at 37 °C and passaged to the second generation when the cell density reached approximately 90% confluence.

### Rat OA model

Male SD rats (9 weeks old, *n* = 90) were anesthetized by administering an intraperitoneal injection of 3% pentobarbital solution (30 mg/kg) and placed on a sterile operating table. A longitudinal incision about 2 cm long was cut in the medial knee joint, and the anterior and posterior cruciate ligament and medial collateral ligament were cut, and the medial meniscus was removed intact without damaging the articular cartilage surface. Postoperative injured limbs are not fixed, and antibiotics can be given to prevent infection. Osteoarthritis forms due to postoperative joint instability, increased friction of the articular surface, and the loss of the buffering effect of the meniscus.

### Experimental grouping

The in vitro experiments were conducted in two parts: the first part: blank, ADAMTS-5 inhibitor, SMAD3 protein and SIS3; the second part: blank, ADAMTS-5 inhibitor, miRNA-140 mimics, None-miRNA-140 mimics (N-miRNA-140 mimics), miRNA-140 inhibitor, None-miRNA-140 inhibitor (N-miRNA-140 inhibitor). The in vivo experiments were conducted in two parts: the first part: blank, ADAMTS-5 inhibitor, SMAD3 protein and SIS3; the second part: blank, ADAMTS-5 inhibitor, miRNA-140 mimics, miRNA-140 inhibitor, random RNA sequence.

### Cell treatment for in vitro experiments

Ultra-clean table sterilization, strict aseptic operation, and the addition of IL-1(interleukin)in the petri dish to induce normal chondrocytes into inflammatory cells, according to experimental groupings, were given corresponding treatment, ordering SMAD3-specific inhibitor SIS3 and miRNA-140 overexpression and inhibition fragments from reagent companies (APExBIO, USA). Cell treatment was performed according to the concentration provided by the reagent company. The concentration of miRNA-140 mimics and miRNA-140 inhibitor was 60 nM, the concentration of SIS3 was 4 nM, the concentration of Smad3 protein was 500 ng, and the concentration of ADAMTS-5 inhibitor in the control group was 2.22 μM. Total RNA was extracted at 24 h, 48 h, and 72 h after treatment for qRT–PCR experiments, and total proteins were extracted at 72 h for Western blot experiments.

### Intra-articular injection for in vivo experiments

After the model rats were grouped, they were intra-articularly injected 1 week after surgery, and the injection was repeated on the tenth day after the operation. Intra-articular injection was performed according to the concentration provided by the reagent company. The total volume of injection was 100 μl, the concentration of SIS3 and SMAD3 protein was 5 μg, miRNA-140 mimics and miRNA-140 inhibitor were lentiviral packaged, the total amount of injection was 1 × 107, and three collected knee specimens were sacrificed in each group at 2 W, 6 W and 12 W after surgery. Total RNA and protein were extracted, and the specimens were selected for fixation and decalcification at each time point in each group.

### Immunohistochemistry and histological staining

The knee joint specimen was soaked in 4% paraformaldehyde at 25 °C for 3 d. After dissection, the muscle was removed, and 20% EDTA was used to decalcify the knee joint for 2 weeks. The knee joint was then embedded in paraffin. The paraffin-fixed tissue sections (5 μm) were baked, dewaxed, and hydrated for ADAMTS-5 (1:200, APExBIO, USA) and Smad3 (1:25, APExBIO, USA) protein immunohistochemistry as well as histological staining using Safranin O/Fast Green and H&E.

### Quantitative reverse transcription polymerase chain reaction (qRT–PCR)

Total RNA was extracted using the TRIzol method at 24 h, 48 h, and 72 h after transfection of the cell samples. The articular cartilage was removed from the tissue samples and ground into a powder after adding liquid nitrogen. RNA was extracted using the TRIzol method, and all experimental steps were carried out on ice. A PCR assay (ABI, USA) was performed using the TaqMan® miRNA reverse transcription kit to reverse transcribe the extracted RNA into complementary DNA (cDNA). For every sample, a final reaction volume of 20 μL, comprising 1 μL of each cDNA and specific primer, was used for the qRT–PCR assay. The thermal cycling conditions were as follows: initial denaturation at 95 °C for 1 min, followed by 40 cycles of 95 °C for 15 s and amplification at 65 °C for 1 min. The 2^-ΔΔCT^ method was used to calculate the relative gene expression. Internal reference: GAPDH, Gene sequences are as follows (5′-3′): miRNA-140 mimics, CAGTGGTTTTACCCTATGGTAG, miRNA-140 inhibitor, CTACCATAGGGTAAAACCACTG, ADAMTS-5, F: GGAGCGAGGCCATTTACAAC, R: CGTAGACAAGGTAGCCCACTTT.

### Western blot

After culturing for 72 h, cell samples were washed twice with PBS, and cell lysates (RIPA: PMSF = 100:1) in approximately 200 μL were obtained. To obtain rat tissue samples from the joint cavity, the lower cartilage tissue was cut using a blade. After adding liquid nitrogen, the tissue samples were ground into powder, followed by the addition of 500 μL cell lysates (RIPA: PMSF = 100:1). The supernatant was centrifuged for 30 min (12,000 XG × 15 min). The protein concentration was measured by using the BCA method, in which 5× loading buffer was added (4:1) to the samples, which were then boiled at 100 °C for 8 min, aliquoted into tubes and frozen until further analysis. The protein samples were separated using SDS–PAGE and transferred onto a PVDF membrane. The membrane was blocked with 5% skimmed milk for 3 h and incubated overnight with ADAMTS-5 primary antibody (1:250; APExBIO, USA) at 4 °C. Subsequently, the membrane was incubated for 2 h in a goat anti-rabbit IgG secondary antibody (1:5000, APExBIO, USA). The membrane was washed and examined under a chemiluminescence instrument. β-actin was used as an internal reference, and gray level analysis was performed using ImageJ software.

### Statistical analysis

The results of this study are all obtained from three or more independent experiments. Measurement data conforming to the normal distribution are described in the mean ± standard deviation, measurement data not conforming to the normal distribution are described in the median and interquartiles. Statistical analysis and the bar graph was made using Graphpad8.0, Adobe Illustrator 2020, Adobe Photoshop 2020 for picture editing, repeated measurement design between groups, multiple comparisons between multiple sample means were performed using the SNK-q test, non-parametric test was used between groups with uneven variance, *P < 0.05* was considered as a statistically significant difference.

## Results

### SIS3 and miRNA-140 mimics significantly inhibited ADAMTS-5 mRNA expression in OA chondrocytes

**Fig. 1:**
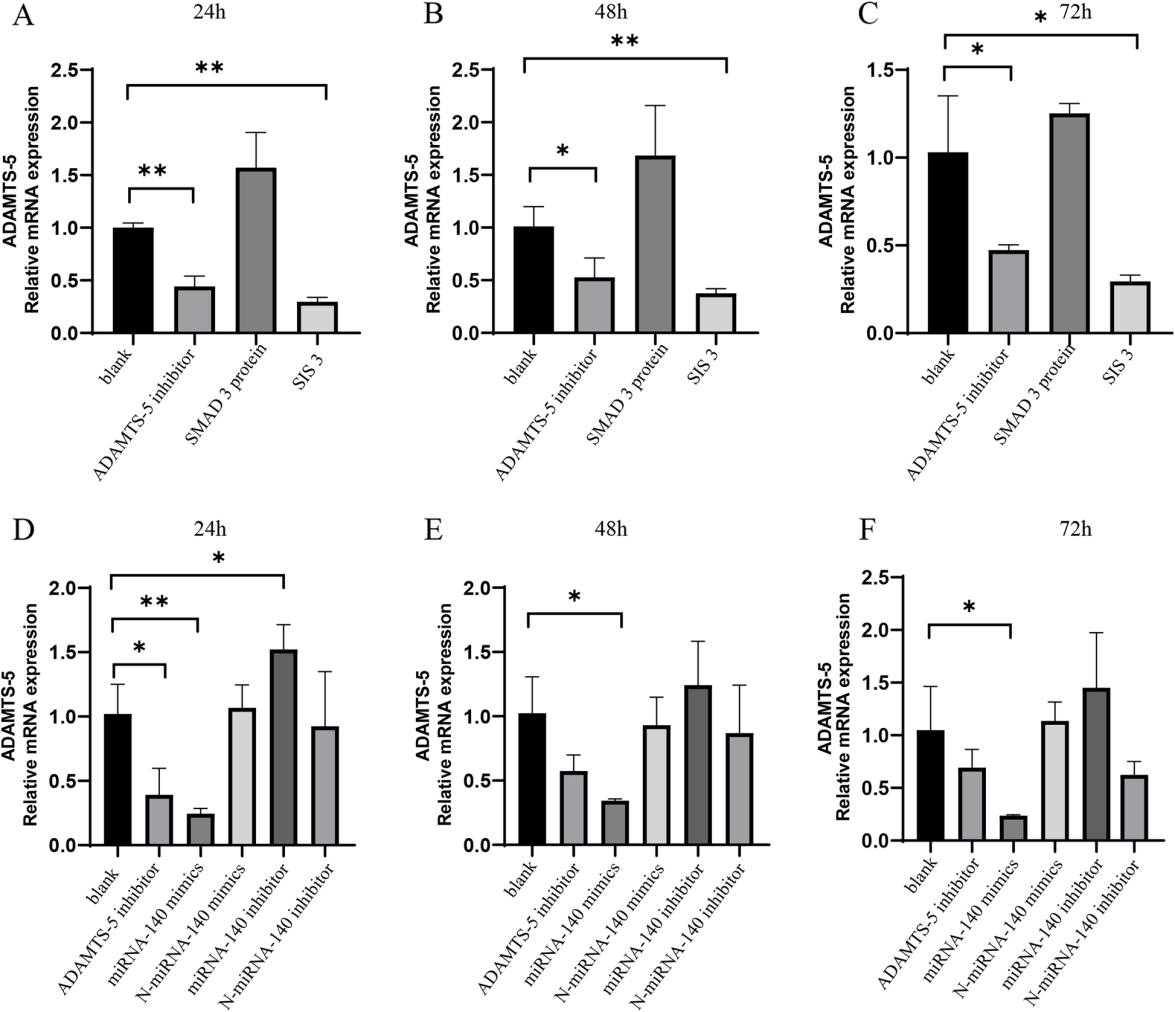
PCR analysis of ADAMTS-5 levels in OA chondrocytes. **A**, **B**, and **C** The relative expression levels of ADAMTS-5 in OA chondrocytes treated with SIS3 at 24, 48, and 72 h. (D, E, and F) The relative levels of ADAMTS-5 in OA chondrocytes treated with miRNA-140 mimics at 24, 48, and 72 h. *, Compared to the blank group, *P* < 0.05. **, Compared to the blank group, *P* < 0.01

To clarify the regulatory effect of the SMAD3 inhibitor (SIS3) and miRNA-140 mimics on ADAMTS-5 expression at the cellular level, qRT–PCR was performed (Fig. 1). At 24 h after cell transfection, ADAMTS-5 mRNA expression was significantly decreased in the ADAMTS-5 inhibitor and SIS3 groups (*P < 0.05*; Fig. 1A). The mRNA expression of ADAMTS-5 was also significantly decreased at 48 and 72 h (*P < 0.05*; Fig. 1B, C). After miRNA-140 overexpression, the mRNA expression of ADAMTS-5 was significantly decreased at 24, 48, and 72 h (*P < 0.05*). After miRNA-140 inhibitor treatment, ADAMTS-5 mRNA expression was significantly upregulated at 24 h (*P < 0.05*) and without any statistical significance at 48 and 72 h (Fig. 1D, E, F).

### SIS3 significantly upregulated the expression of miRNA-140 in OA chondrocytes

**Fig. 2:**
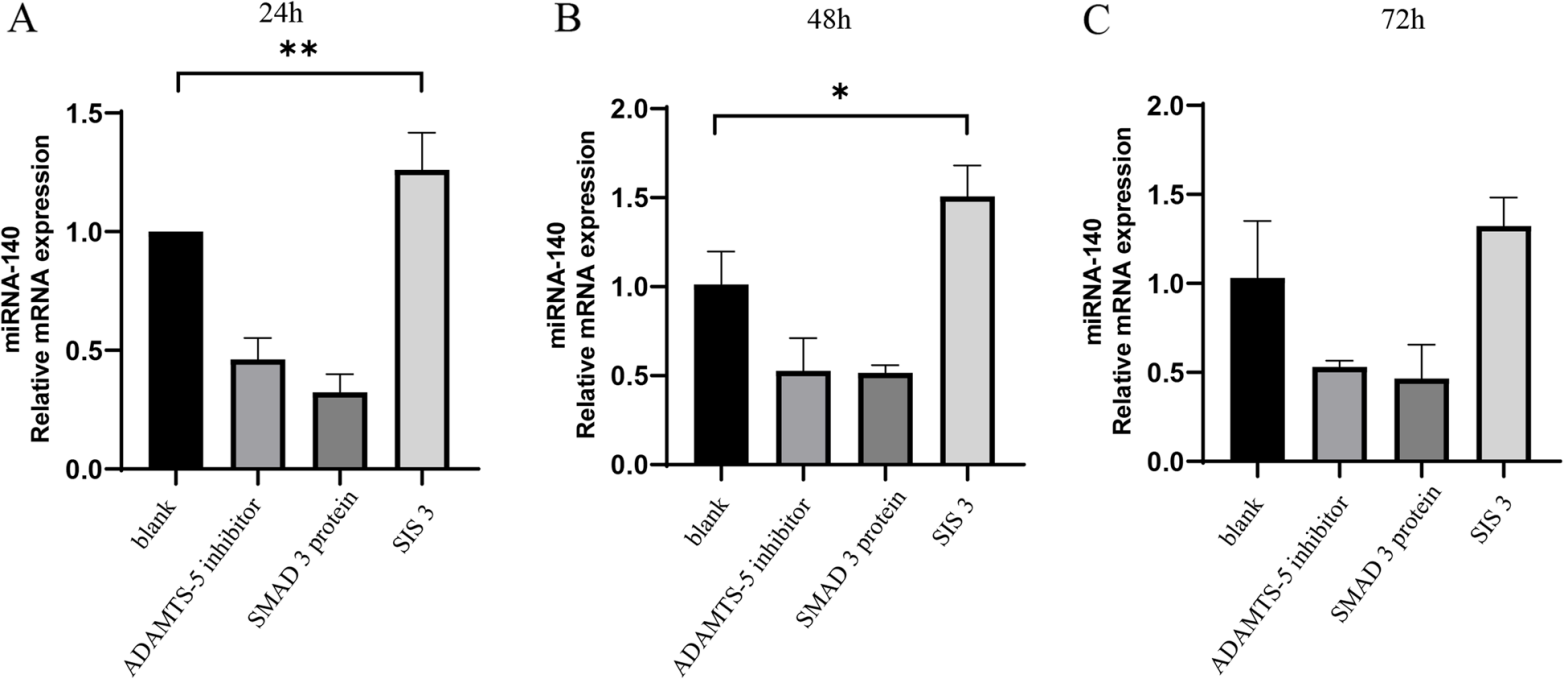
PCR analysis of miRNA-140 levels in OA chondrocytes treated with SIS3 at 24, 48, and 72 h. *, Compared to the blank group, *P* < 0.05. **, Compared to the blank group, *P* < 0.01

We detected the cellular-level expression of miRNA-140 after SIS3 treatment (Fig. 2). The expression of miRNA-140 in the SIS3 group was upregulated to varying degrees at 24 and 48 h, with statistical significance (*P < 0.05*; Fig. 2A, B), except at 72 h.

### SIS3 and miRNA-140 mimics significantly inhibited ADAMTS-5 mRNA expression in OA cartilage tissue

**Fig. 3:**
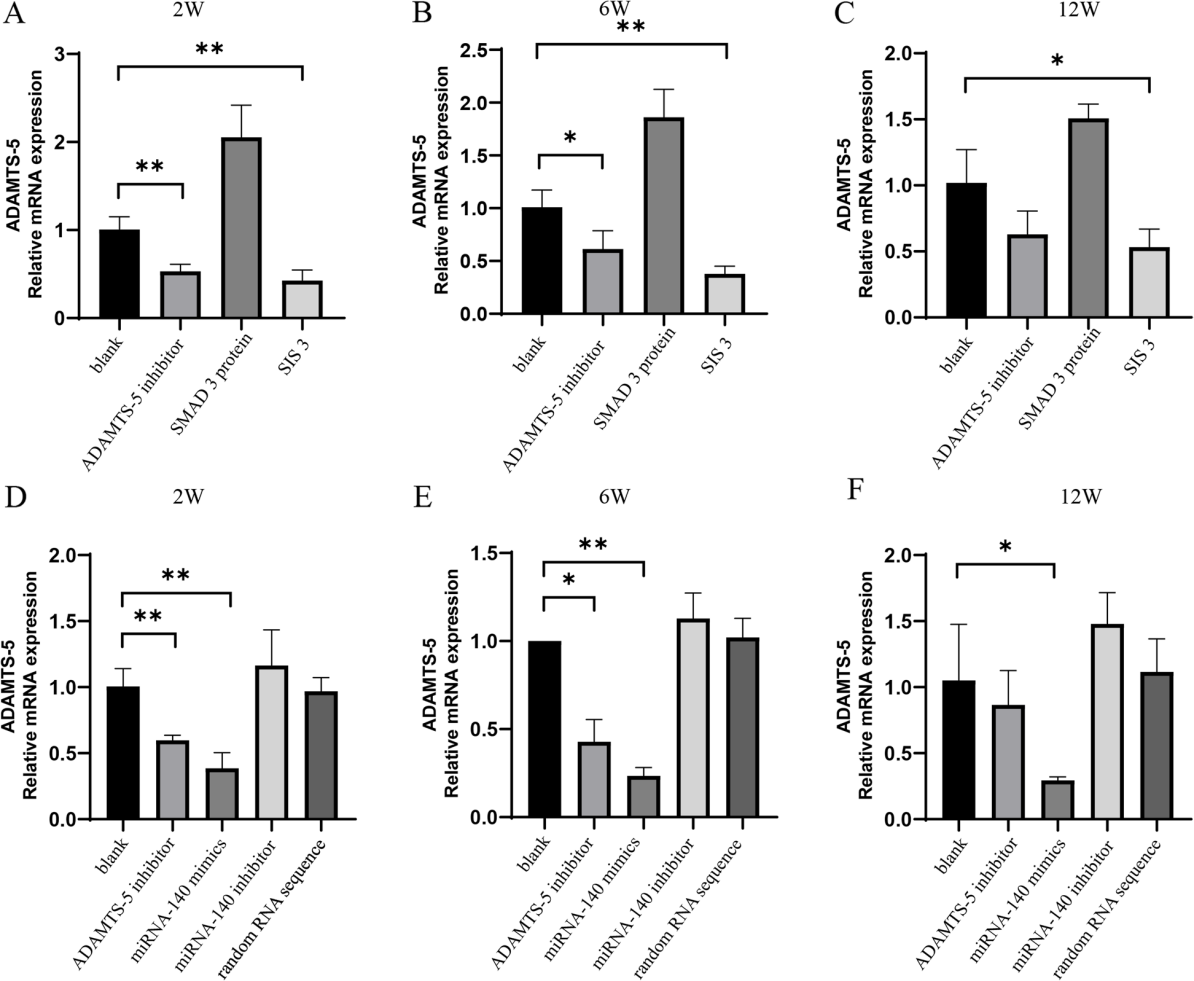
PCR analysis of ADAMTS-5 levels in OA cartilage tissue after intra-articular injection of SIS3 and miRNA-140 mimics. **A**, **B**, and **C** The relative expression levels of ADAMTS-5 in OA cartilage tissue after intra-articular injection of SIS3 at 2, 6, and 12 weeks. (D, E, and F) The relative expression levels of ADAMTS-5 in OA cartilage tissue after intra-articular injection of miRNA-140 mimics at 2, 6, and 12 weeks. *, Compared to the blank group, *P* < 0.05. **, Compared to the blank group, *P* < 0.01. ns, Compared to the blank group, *P* > 0.05

Subsequently, the in vivo regulatory effect of SIS3 and miRNA-140 mimics on ADAMTS-5 at the gene level was verified by intra-articular injection (Fig. 3). The mRNA expression of ADAMTS-5 was significantly downregulated at 2 and 6 weeks after intra-articular injection of SIS3 (*P < 0.05*) (Fig. 3A, B) but not at 12 weeks (Fig. 3C). The expression of ADAMTS-5 mRNA was significantly downregulated after miRNA-140 mimic treatment at 2 weeks (*P < 0.05*) (Fig. 3D) and without any statistical significance at 6 and 12 weeks (Fig. 3E, F).

### SIS3 significantly upregulated the expression of miRNA-140 in OA cartilage tissue

**Fig. 4:**
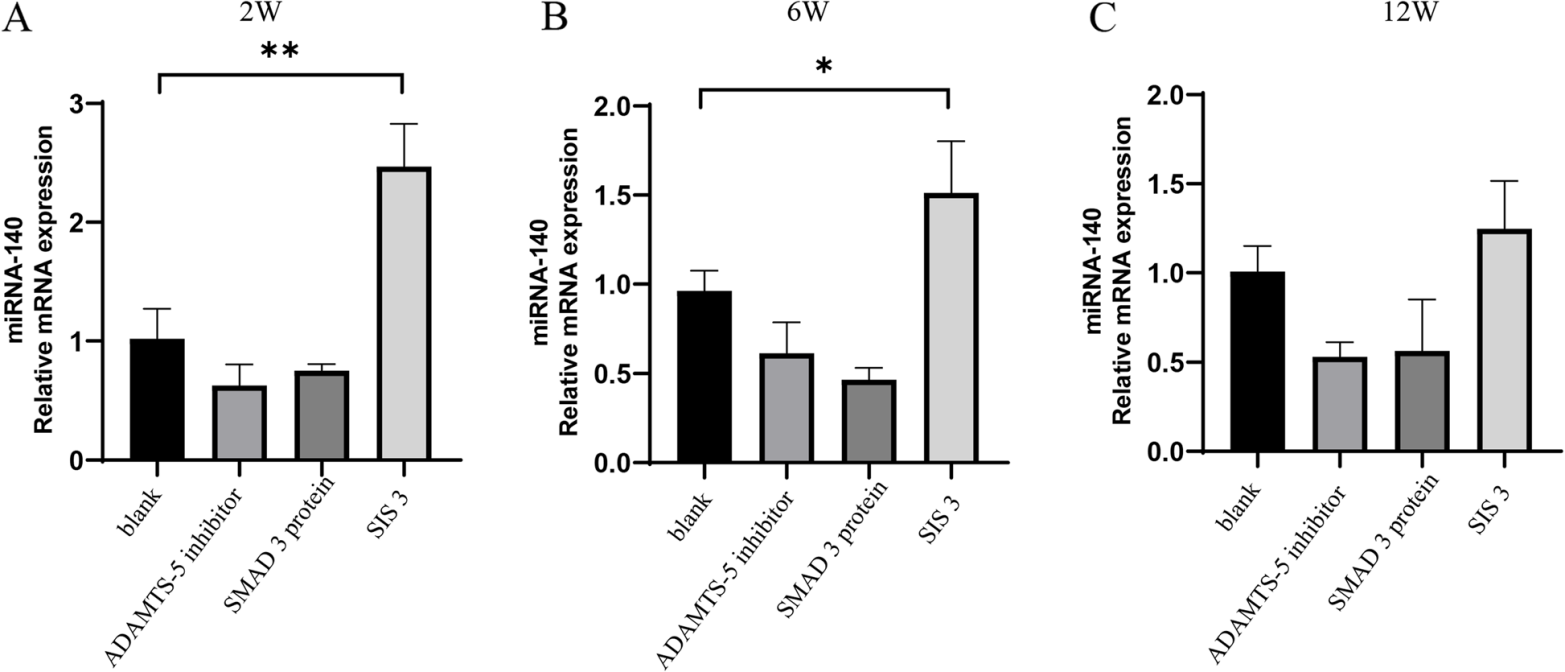
PCR analysis of miRNA-140 levels in OA cartilage tissue after intra-articular injection of SIS3 at 2, 6, and 12 weeks. *, Compared to the blank group, *P* < 0.05. **, Compared to the blank group, *P* < 0.01

In vivo expression of miRNA-140 was also detected after injection of SIS3 (Fig. 4). The expression of miRNA-140 was significantly increased at all three time points with statistical significance at 2 and 6 weeks (*P < 0.05*) (Fig. 4A, B), but not at 12 weeks (Fig. 4C).

### SIS3 and miRNA-140 mimics significantly inhibited ADAMTS-5 protein expression in OA chondrocytes

**Fig. 5:**
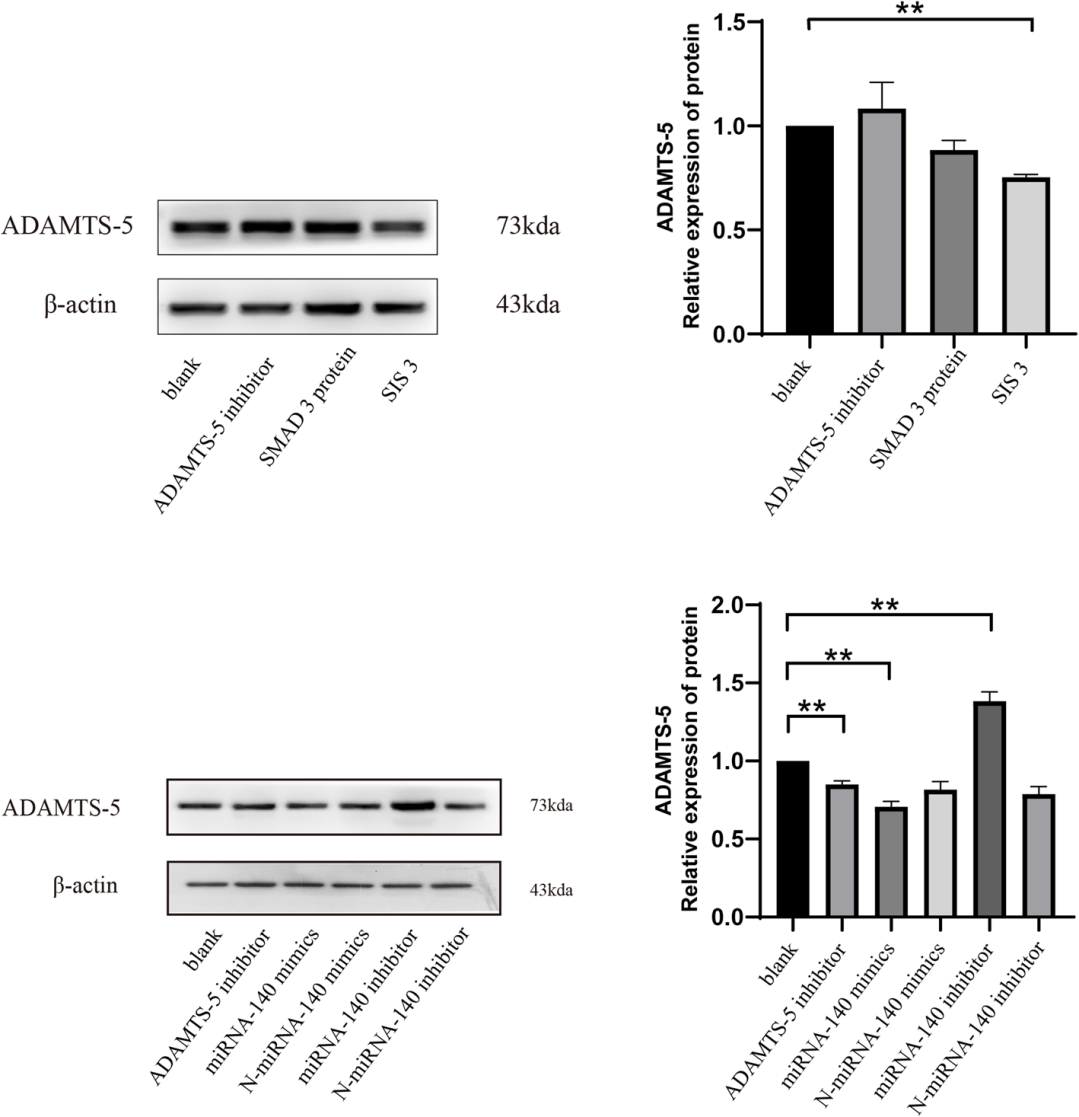
Protein expression of ADAMTS-5 in OA chondrocytes treated with SIS3 and miRNA-140 mimics at 72 h. *, Compared to the blank group, *P* < 0.05. All the gels were trimmed and the samples derive from the same experiment and that gels/blots were processed in parallel

After 72 h of cell culturing, protein was extracted for western blot analysis (Fig. 5). At the protein level, the expression of ADAMTS-5 in the SIS3 and miRNA-140 mimic groups was significantly decreased (*P < 0.05*), while the expression of ADAMTS-5 protein in the miRNA-140 inhibitor group was significantly upregulated (*P < 0.05*).

### SIS3 and miRNA-140 mimics significantly inhibited ADAMTS-5 protein expression in OA cartilage tissue

**Fig. 6:**
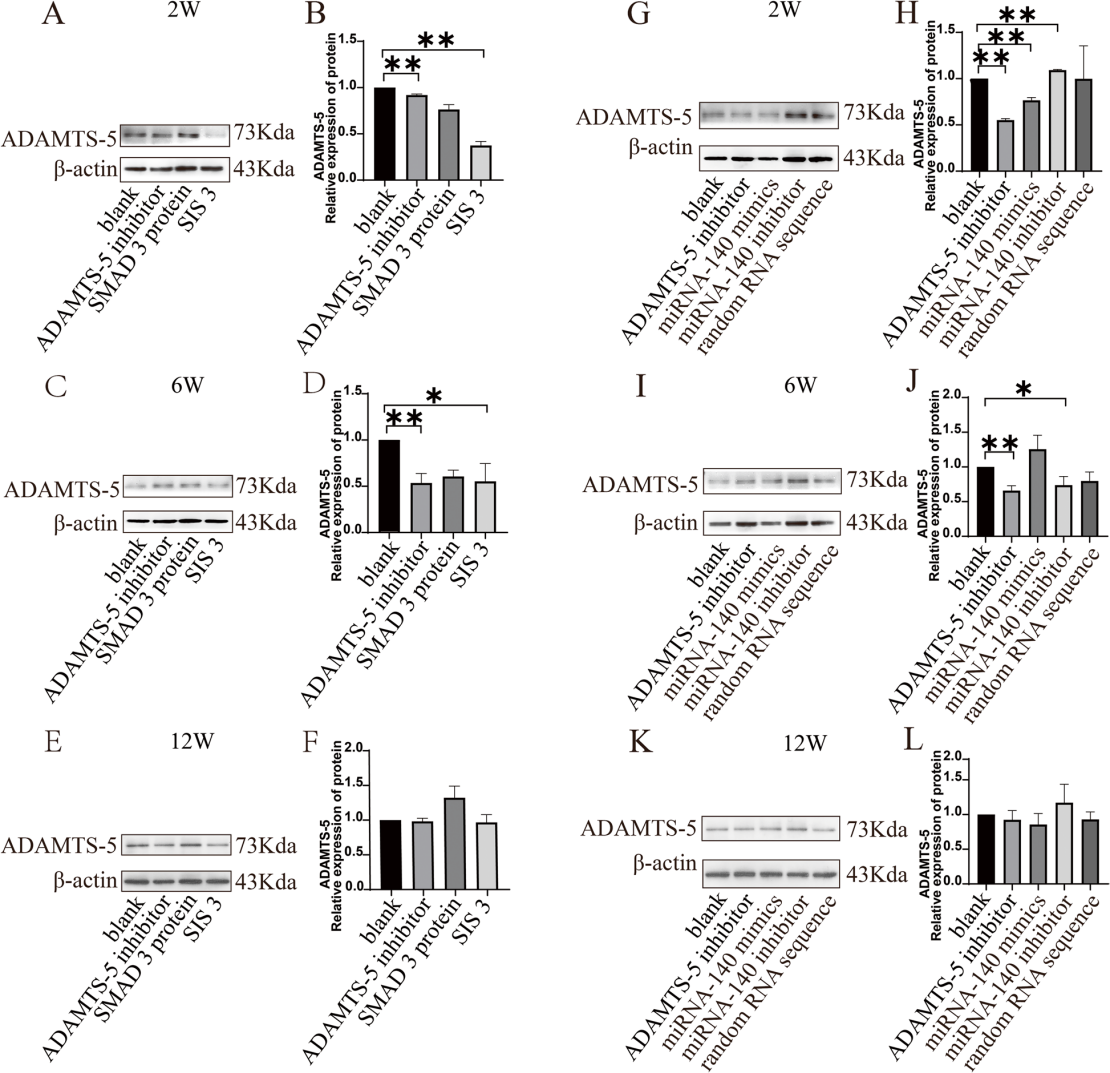
ADAMTS-5 expression in OA cartilage tissue after intra-articular injection of SIS3 and miRNA-140 mimics. **A**, **B** ADAMTS-5 expression at 2 weeks. **C**, **D** ADAMTS-5 expression at 6 weeks. **E**, **F** ADAMTS-5 expression at 12 weeks. **G**, **H** ADAMTS-5 expression at 2 weeks. (I, J) ADAMTS-5 expression at 6 weeks. (K, L) ADAMTS-5 expression at 12 weeks. *, Compared to the blank group, *P* < 0.05. **, Compared to the blank group, *P* < 0.01.All the gels were trimmed and the samples derive from the same experiment and that gels/blots were processed in parallel

The in vivo regulatory effect of SIS3 on ADAMTS-5 at the protein level was verified by intraarticular administration (Fig. 6). The expression of the target protein at 2 weeks was significantly decreased (*P < 0.05*), and the difference at 6 weeks was not as profound as that of week 2, and there was almost no difference by 12 weeks (Fig. 6A-F). The expression of target proteins in the miRNA-140 mimic group decreased significantly at 2 weeks (*P < 0.05*) (Fig. 6G, H) and without any statistical significance at 6 and 12 weeks (Fig. 6I-L).

### HE staining revealed a relative reduction of cartilage degeneration in the SIS3 and miRNA-40 mimics groups

**Fig. 7:**
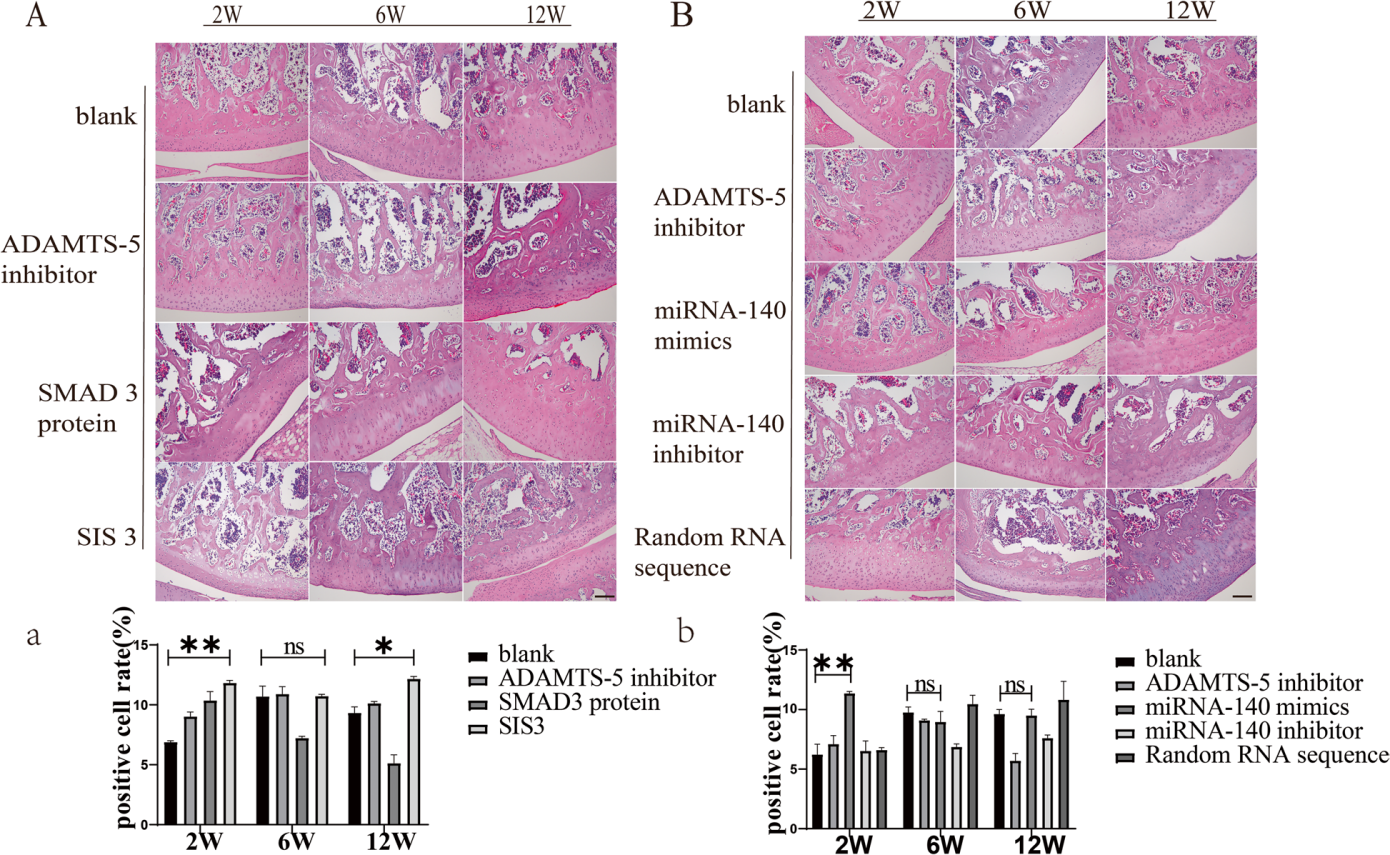
**A**, **B** H&E staining of cartilage tissue after intra-articular injection of SIS3 and miRNA-140 mimics. **C**, **D** Results were quantitatively analysis by HE staining. Scale bar, 100 μm

The results of H&E staining for paraffin-fixed sections of specimens showed that there was no significant change in the cartilage structure of the early SIS3 and miRNA-140 groups compared to the other groups, but a rough cartilage surface and a reduced number of chondrocytes were observed after 6 weeks (Fig. 7). Quantitative analysis of chondrocyte number of H&E results showed that the proportion of chondrocytes in different groups at different time, positive cell rate in SIS3 group compared with the blank group, 2 W and 12 W difference is statistically significant (*P < 0.05*), positive cell rate in miRNA-140 mimics group is also larger than the blank group, 2 W difference is statistically significant (*P < 0.05*).

### ADAMTS-5 immunohistochemistry found a relative reduced expression in the SIS3 and miRNA-140 mimics groups

**Fig. 8:**
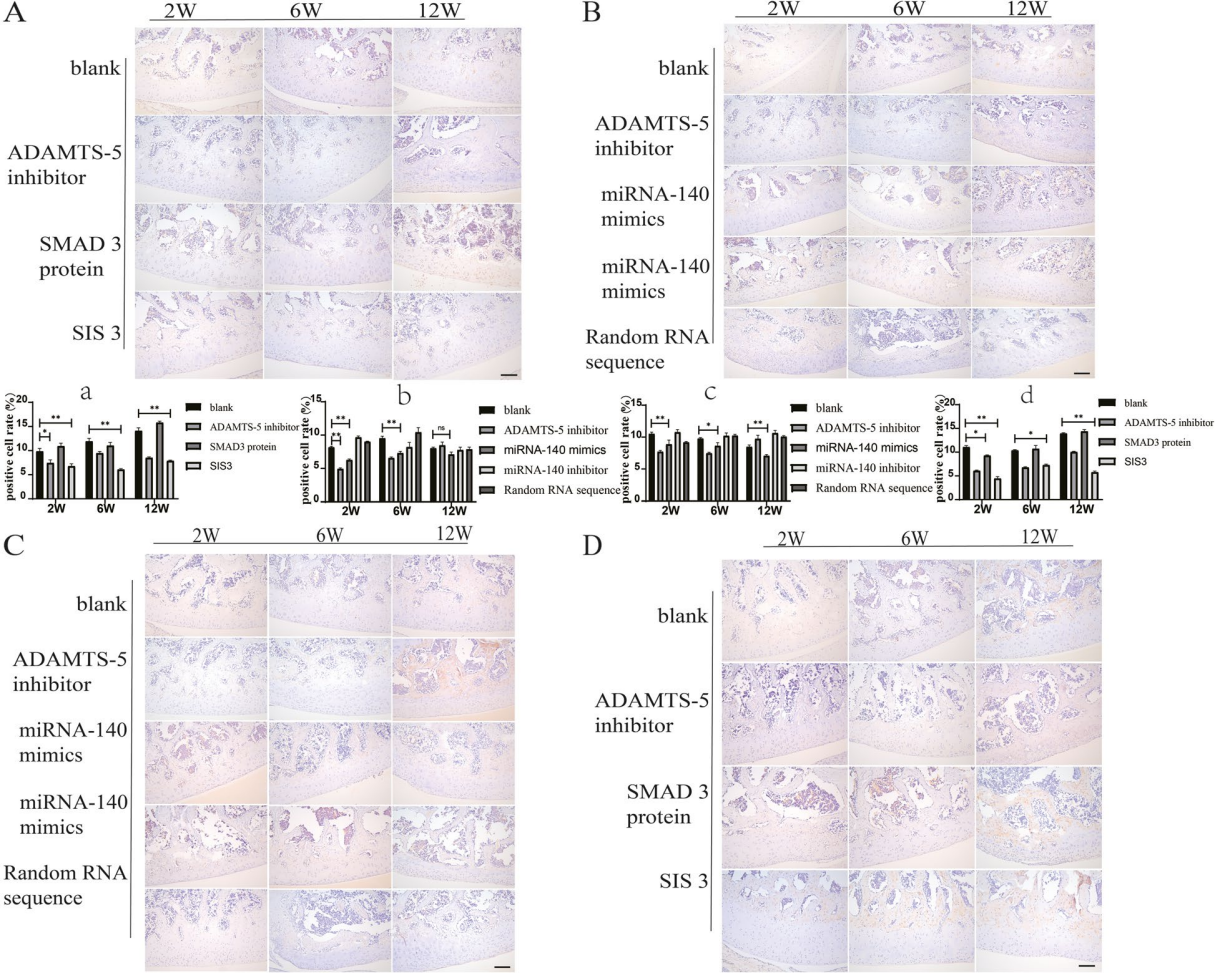
Immunohistochemical analyses for ADAMTS-5 and SMAD3 in cartilage tissue after intra-articular injection of SIS3 and miRNA-140 mimics. **A**, **B** Immunohistochemical analysis of ADAMTS-5. **C**, **D** Immunohistochemical analysis of SMAD3. Scale bar, 100 μm

Immunohistochemical analyses for ADAMTS-5 and Smad3 proteins were performed on paraffin-fixed sections of specimens (Fig. 8). Compared to the blank group, the expression of ADAMTS-5 protein in the SIS3 group was significantly inhibited, the cartilage surface was smooth, and the number of chondrocytes was not significantly reduced. ADAMTS-5 protein was also significantly inhibited in the miRNA-140 mimic group, while high ADAMTS-5 protein expression was detected in the positive control and miRNA-140 inhibitor groups (Fig. 8A, B). To confirm the effectiveness of intra-articular injection of SIS3, the expression level of SMAD3 was also examined. The results showed that the expression level of SMAD3 was significantly decreased in the SIS3 group, while SMAD3 protein was also significantly inhibited in the miRNA-140 mimic group. SMAD3 protein was highly expressed in the positive control and miRNA-140 inhibitor groups (Fig. 8C, D). The immunohistochemical results were quantified, respectively, and the rate of ADAMTS-5 positive cells was significantly reduced in the SIS3 and miRNA-140 mimics groups, which was statistically significant (*P < 0.05*).

### Safranin O/fast green staining revealed a relative reduction of cartilage degeneration in the SIS3 and miRNA-40 mimics groups

**Fig. 9:**
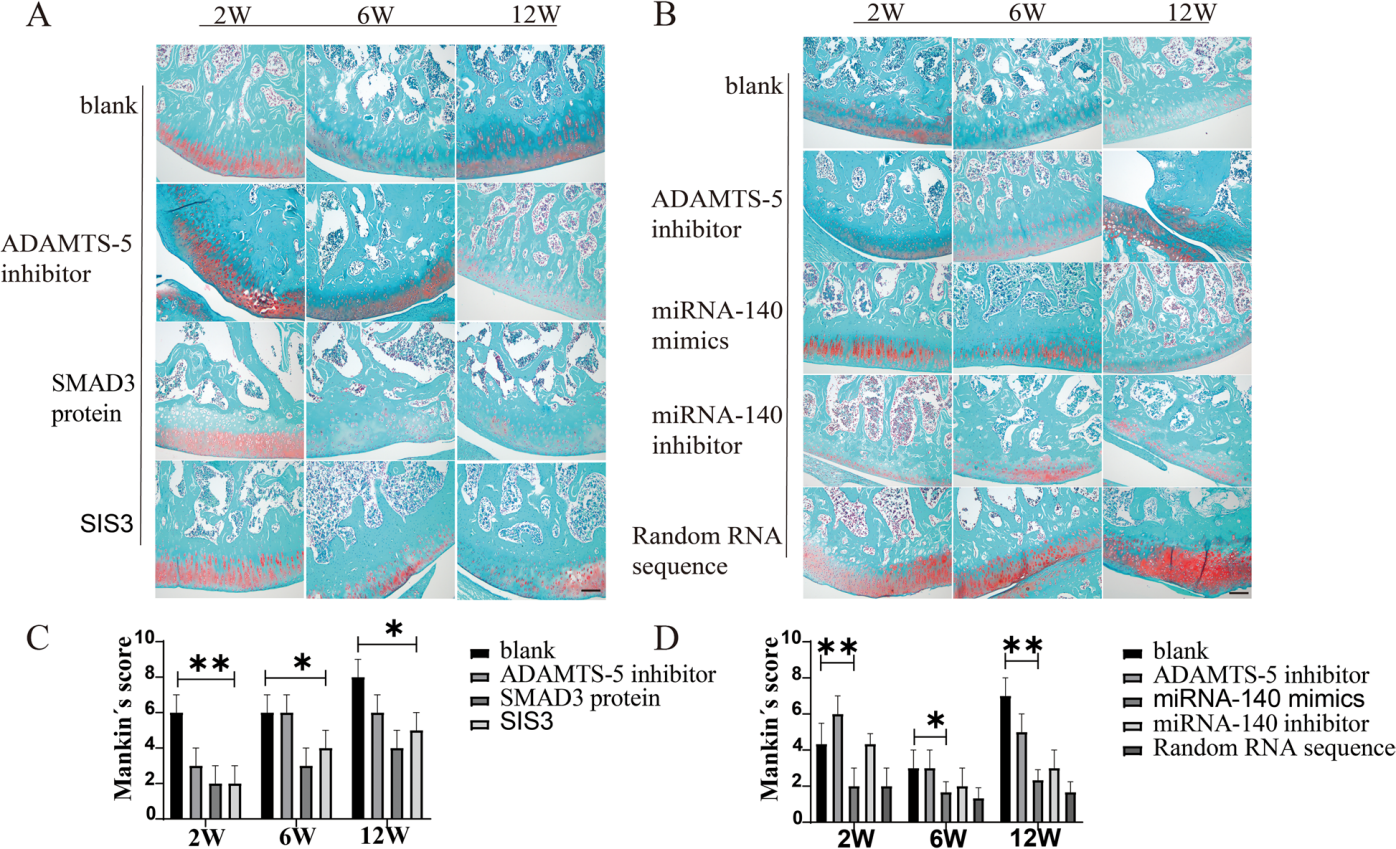
Safranin O/Fast Green staining of cartilage tissue after intra-articular injection of SIS3 and miRNA-140 mimics in OA rats

The results for the severity of cartilage degeneration showed that in the early stage of the SIS3 and miRNA-140 mimic groups, the cartilage surface was smooth and without irregular cracks (Fig. 9). The number of chondrocytes was not significantly reduced, and the tide line was complete. However, progressive degenerative changes began to appear at 6 and 12 weeks, with irregular cracks on the cartilage surface. A large number of clustered cells appeared, and the tide line became blurred due to irregular subchondral blood vessels. Mankin’s score showed that SIS3 and miRNA-140 groups were lower than other groups (*P < 0.05*).

## Discussion

OA is a complex disease characterized by deterioration in the structure, function, and metabolism of the entire joint and peri-joint tissue and is characterized by irreversible cartilage damage, inflammation, and chondrocyte phenotypic changes [30]. Numerous meta-analyses have shown a significant association between SMAD3 gene polymorphisms and OA risk and significant differences in different populations [31–35]. SMAD3 is involved in the TGFβ signal transduction pathway, which has an inhibitory effect on TGF-induced chondrocyte maturation [36, 37], and TGFβ is a homeostatic regulator of subchondral and articular cartilage. There is growing evidence that alterations in TGFβ signaling are involved in the pathogenesis of OA [21, 38, 39] and that members of the TGFβ family can activate multiple intracellular signaling pathways, which are divided into receptor SMAD (R-SMAD)-dependent or R-SMAD-independent types, but the R-SMAD-dependent signaling pathway is considered to be a typical signaling pathway for members of the TGFβ family [40, 41], and the SMAD3 gene is an intron variant. Playing an important role in bone remodeling and cartilage maintenance, high expression of SMAD3 in cartilage degeneration in the knee and hip joints has been confirmed [23]. Therefore, this study selected SIS3, a specific inhibitor of SMAD3, as the main treatment factor of the experiment and observed whether inhibiting the expression of SMAD3 would have a certain impact on the progression of OA.

Gene silencing in OA chondrocytes by transient transfection of specific small interfering RNAs (siRNAs) has identified the binding site of the miRNA-140 regulatory sequence in OA chondrocytes, and SMAD3 can directly regulate the binding site of miR-140 [40], which was also verified by the joint cavity injection of miRNA-40 packaged by lentivirus.

Aggrecanase (A disintegrin and metalloproteinase with thrombospondin motifs, ADAMTS) is a member of the newly cloned detangulating protein metalloproteinase family in recent years, with platelet thrombin-sensitive proteoid modal regions that bind to and participate in ECM metabolism [42, 43]. There are currently no approved drugs (DMOADs) for the treatment of osteoarthritis, and ADAMTS-5 is a promising target for identifying DMOADs [44]. GLPG1972/S201086 is a desyin and metalloproteinase with a platelet-reactive protein motif-5 (ADAMTS-5) inhibitor that is under development as a modified therapy for OA disease [45]. The ADAMTS-5 inhibitor group was also added as a positive control group in this study, and the results showed that the inhibitors could significantly inhibit the expression of ADAMTS-5. Removing ADAMTS-5 activity in vitro and in vitro can combat the loss of aggrecan damage and protect cartilage.

Using ADAMTS-4 and ADAMTS-5 single-gene knockout mice, it was found that compared with wild-type mice, gene knockout animal models showed a significant delay in the process of cartilage degeneration, of which ADAMTS-5 was the key enzyme for proteoglycan degradation in OA articular cartilage in mice [46], so ADAMTS-5 played an important pathological role in early cartilage degeneration [47–49]. In this study, ADAMTS-5 was selected as the main research indicator, and we found that the change in ADAMTS-5 was more obvious at an early time point. In a short-term cartilage culture model in vitro, chondrocytes were stimulated with IL-1, and it was found that Aggrecanase was the main enzyme that degraded enzymes in the first 2 weeks. After approximately 3 weeks of incubation, the degradation of MMP-dependent polypolysaccharide core proteins could be detected, at which point collagen degradation also began [50]. Mort [51] applied imprinted proteins to knee specimens undergoing joint replacement to check the expression of Aggrecanase and MMPs, and the results showed higher expression of MMPS in late joint specimens, which speculated that the degradation of Aggrecanase-mediated occurred in the early stages of OA, while the degradation of MMP-mediated collagen occurred in the subsequent multistage. In our results, we also found that the change at 2 W was more pronounced than in other groups. One studies [42] have shown that in OA articular cartilage, the expressions of ADAMTS-4/5 gene is obvious, and ADAMTS-5 mRNA is significantly upregulated compared with ADAMTS-4 mRNA, which may have a stronger pathological effect on ADAMTS-5 in OA lesions. Studies in recent years [52] have shown that in early joint fluid in patients with OA, polyprotenan cleavage fragments produced by a large number of polyproteranases can be seen, and in early degenerative cartilage, its expression is mainly concentrated in the area of polyprotenan decay. Chu et al [53] evaluated the role of ADAMTS-5 in OA in rat models using siRNA transfection techniques, and the results showed that inhibition of ADAMTS-5 reduced the degradation of polyprotenans in cytokine-stimulated normal cartilage. Delaying the progression of OA by inhibiting the expression of ADAMTS-5 in cartilage is currently a research hotspot.

Local intra-articular injection of vascular endothelial growth factor has been reported to accelerate articular cartilage degeneration in rat osteoarthritis models [54], and clinical trials of intra-articular injection of autologous fat mesenchymal stem cells for knee osteoarthritis have been reported [55, 56]. This study also used the articular injection method to explore the regulatory relationship between SMAD3 and ADAMTS-5 expression in in vitro experiments according to the recommended dose, and the postoperative 2 W ADAMTS-5 was significantly downregulated at the gene and protein levels, but with the passage of time, the difference became less obvious, which may be related to drug concentration and metabolic inactivation. At the same time, we used lentiviral-packaged miRNA-140 mimics and inhibitors to verify the regulatory relationship between miRNA-140 and ADAMTS-5, and the results showed that there was also a negative regulatory relationship between the two, and the overexpression of miRNA-140 could downregulate the expression of ADAMTS-5, which was consistent with a previous literature report [28].

In our study, we found that in the early stage of OA, SIS3 and miRNA-140 mimics can significantly delay cartilage degeneration, the number of chondrocytes is not significantly reduced, and the tidal line is intact. SMAD3 is able to regulate ADAMTS-5 expression, and the two are a positive regulatory relationship.

This study innovatively uses the method of intra-articular injection of SIS3 and miRNA-140 mimics, breaking the traditional method of administration through blood vessels and intraperitoneal injection, so that it acts directly on cartilage in the joint cavity, which can reduce the catabolism and ensure the high concentration of drugs. The limitation of this study is that it only pays attention to the changes of early OA, fails to repeatedly carry out intra-articular injection to observe the progress of mid- and late OA, because there will be metabolic inactivation and concentration reduction of intra-articular injection drugs and lentivirus. Effective utilization cannot be guaranteed, and future experiments need to be improved.

## Conclusion

Inhibition of SMAD3 can upregulate the expression of miRNA-140, which in turn inhibits the expression of ADAMTS-5. Concurrently, SMAD3 inhibition can reduce the in vivo and in vitro expression of ADAMTS-5, which has been confirmed at both the protein and gene levels. Our study demonstrated that the expression of target genes and proteins at both the protein and gene levels changed significantly at the early time point; hence, we inferred that SMAD3 could mediate miRNA-140 to regulate the expression of ADAMTS-5 in the early stages of OA, and in-depth studies are vital to reveal the specific regulatory mechanism of OA pathogenesis and provide therapeutic strategies for the clinic.

## Supplementary Information

**Additional file 1.**

## Data Availability

All data generated or analysed during this study are included in this published article and its supplementary information files.
