# Correction: Inhibition of SMAD3 effectively reduces ADAMTS‑5 expression in the early stages of osteoarthritis

**DOI:** 10.1186/s12891-023-06262-8

**Published:** 2023-03-06

**Authors:** Wei Xiang, Chao Wang, Zhoujun Zhu, Dui Wang, Zhenyu Qiu, Weishan Wang

**Affiliations:** 1 Renmin Hospital of Zhijiang, Yichang, Hubei China; 2 Department of Orthopedics Center, The First Affiliated Hospital, Shihezi University School of Medicine, 107 North Second Road, 832000 Shihezi, Xinjiang People’s Republic of China; 3 Shihezi University School of Medicine, Xinjiang, China; 4 Department of Joint Surgery, The Sixth Affiliated Hospital of Xinjiang Medical University, Urumqi, Xinjiang Uygur Autonomous Region China

**Correction: BMC Musculoskelet Disorders 24, 130 (2023)**

10.1186/s12891-022-05949-8

Following publication of the original article [1], the authors identified an error in the first author’s affiliation. Dr. Wei Xiang’s affiliation should only be “^1^Renmin Hospital of Zhijiang, Yichang, Hubei, China”.

The original article [1] has been updated.
