# Supplementary material for: Inhibition of SMAD3 effectively reduces ADAMTS-5 expression in the early stages of osteoarthritis
Source: BMC Musculoskelet Disord. 2023 Feb 17;24:130. doi: 10.1186/s12891-022-05949-8 (PMC9936734; doi:10.1186/s12891-022-05949-8)
Supplement: Supplementary file 1 — Additional file 1. [file 12891_2022_5949_MOESM1_ESM.zip › Figure legend.docx]

Fig. 1. PCR analysis of ADAMTS-5 levels in OA chondrocytes. (A, B, and C) The relative expression levels of ADAMTS-5 in OA chondrocytes treated with SIS3 at 24, 48, and 72 h. (D, E, and F) The relative levels of ADAMTS-5 in OA chondrocytes treated with miRNA-140 mimics at 24, 48, and 72 h. *, Compared to the blank group, P < 0.05. **, Compared to the blank group, P < 0.01.

Fig. 2. PCR analysis of miRNA-140 levels in OA chondrocytes treated with SIS3 at 24, 48, and 72 h. *, Compared to the blank group, P < 0.05. **, Compared to the blank group, P < 0.01.

Fig. 3. PCR analysis of ADAMTS-5 levels in OA cartilage tissue after intra-articular injection of SIS3 and miRNA-140 mimics. (A, B, and C) The relative expression levels of ADAMTS-5 in OA cartilage tissue after intra-articular injection of SIS3 at 2, 6, and 12 weeks. (D, E, and F) The relative expression levels of ADAMTS-5 in OA cartilage tissue after intra-articular injection of miRNA-140 mimics at 2, 6, and 12 weeks. *, Compared to the blank group, P < 0.05. **, Compared to the blank group, P < 0.01. ns, Compared to the blank group, P > 0.05.

Fig. 4. PCR analysis of miRNA-140 levels in OA cartilage tissue after intra-articular injection of SIS3 at 2, 6, and 12 weeks. *, Compared to the blank group, P < 0.05. **, Compared to the blank group, P < 0.01.

Fig. 5. Protein expression of ADAMTS-5 in OA chondrocytes treated with SIS3 and miRNA-140 mimics at 72 h. *, Compared to the blank group, P < 0.05. All the gels were trimmed and the samples derive from the same experiment and that gels/blots were processed in parallel.

Fig. 6. ADAMTS-5 expression in OA cartilage tissue after intra-articular injection of SIS3 and miRNA-140 mimics. (A, B) ADAMTS-5 expression at 2 weeks. (C, D) ADAMTS-5 expression at 6 weeks. (E, F) ADAMTS-5 expression at 12 weeks. (G, H) ADAMTS-5 expression at 2 weeks. (I, J) ADAMTS-5 expression at 6 weeks. (K, L) ADAMTS-5 expression at 12 weeks. *, Compared to the blank group, P < 0.05. **, Compared to the blank group, P < 0.01.All the gels were trimmed and the samples derive from the same experiment and that gels/blots were processed in parallel.

Fig. 7.(A, B)H&E staining of cartilage tissue after intra-articular injection of SIS3 and miRNA-140 mimics. (C, D) Results were quantitatively analysis by HE staining.Scale bar, 100 µm.

Fig. 8. Immunohistochemical analyses for ADAMTS-5 and SMAD3 in cartilage tissue after intra-articular injection of SIS3 and miRNA-140 mimics. (A, B) Immunohistochemical analysis of ADAMTS-5. (C, D) Immunohistochemical analysis of SMAD3. Scale bar, 100 µm.

Fig. 9. Safranin O/Fast Green staining of cartilage tissue after intra-articular injection of SIS3 and miRNA-140 mimics in OA rats.
