# Supplementary material for: Inhibition of SMAD3 effectively reduces ADAMTS-5 expression in the early stages of osteoarthritis
Source: BMC Musculoskelet Disord. 2023 Feb 17;24:130. doi: 10.1186/s12891-022-05949-8 (PMC9936734; doi:10.1186/s12891-022-05949-8)
Supplement: Supplementary file 1 — Additional file 1. [file 12891_2022_5949_MOESM1_ESM.zip › raw data(WB+IHC+HE+Safranin O stain).docx]

# WB


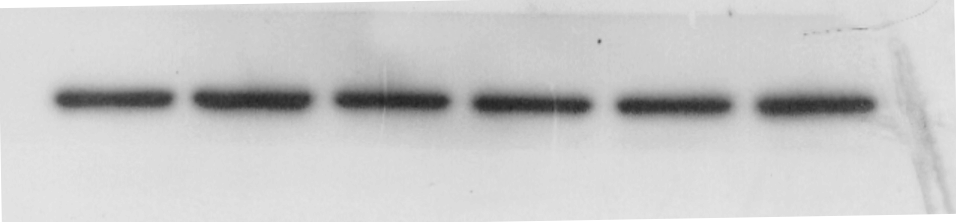


cell sample-WB-β-actin (miRNA-140)


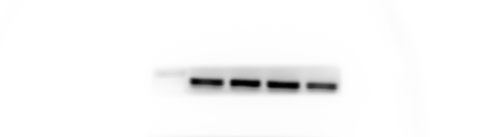


cell sample-WB-ADAMTS-5（SIS3）


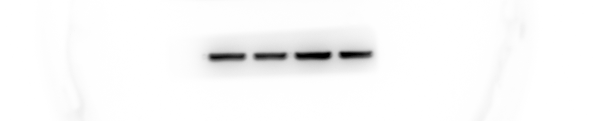


cell sample-WB-β-actin （blank-ADAMTS-5 inhibitor-SMAD3 protein-SIS3）


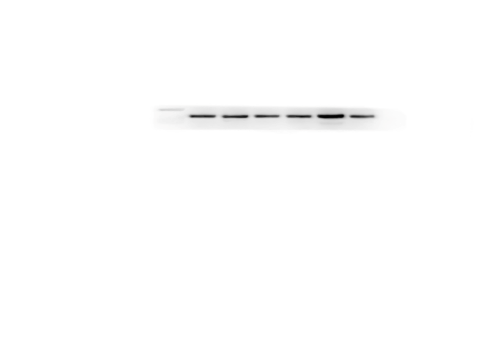


cell sample-WB-ADMTS-5(miRNA-140 mimics)


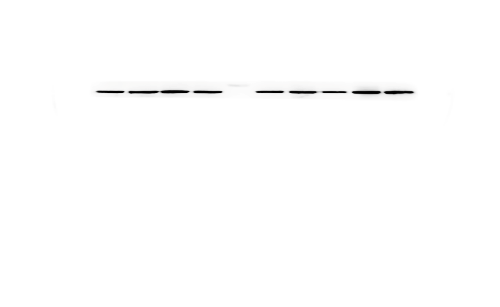


tissue sample-WB-β-actin-2 week（left：SIS3，right：miRNA-140）


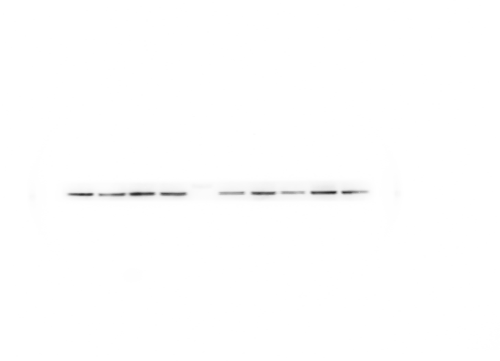


tissue sample-WB-β-actin-6 week（left：SIS3，right：miRNA-140）


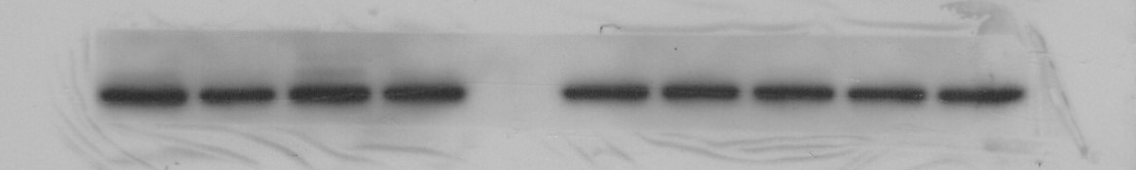


tissue sample-WB-β-actin-12 week（left：SIS3，right：miRNA-140）


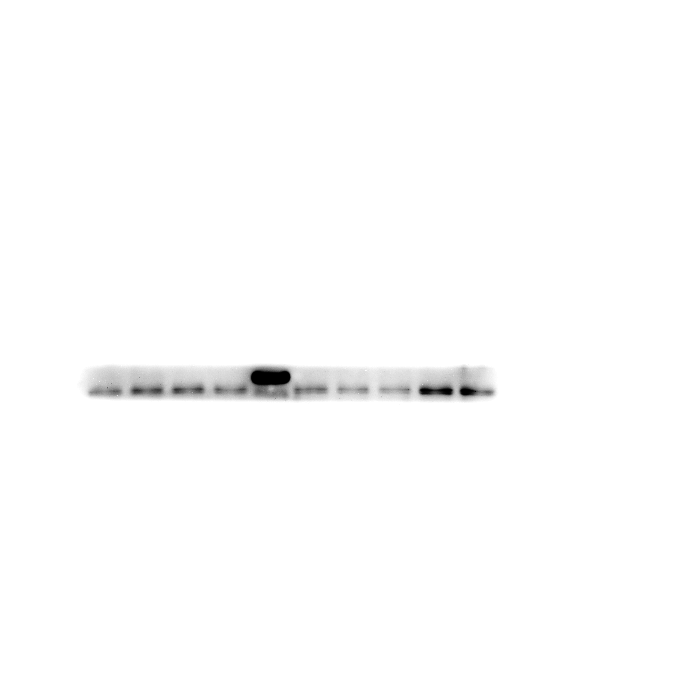


tissue sample-WB-ADAMTS-5-2 week（left：SIS3，right：miRNA-140）





tissue sample-WB-ADAMTS-5-6 week（left：SIS3，right：miRNA-140）


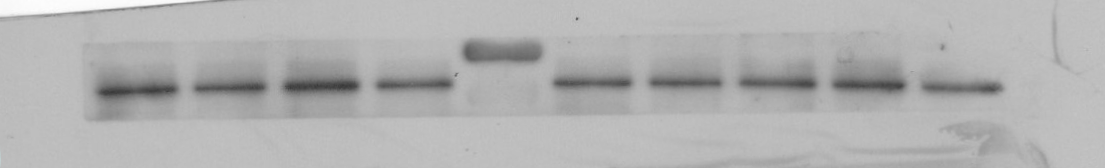


tissue sample-WB-ADAMTS-5-12 week（left：SIS3，right：miRNA-140）

# HE


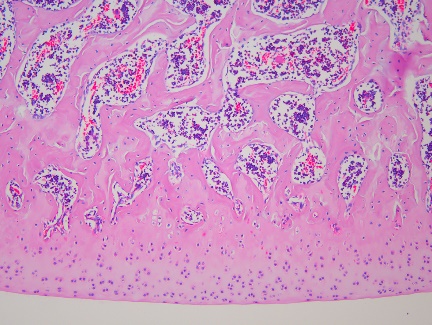


HE-A-ADAMTS-5 inhibitor-2 Week


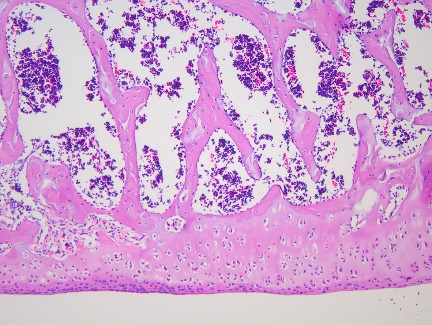


HE-A-ADAMTS-5 inhibitor-6 Week


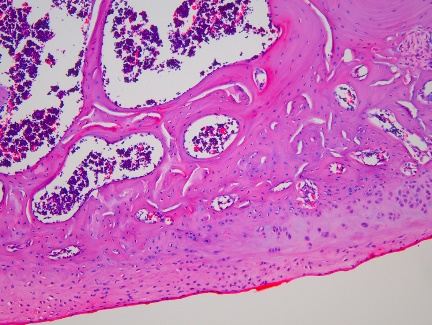


HE-A-ADAMTS-5 inhibitor-12 Week


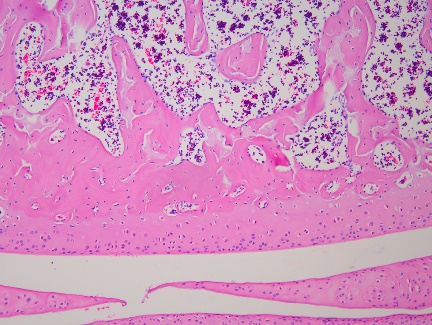


HE-A-blank-2 week


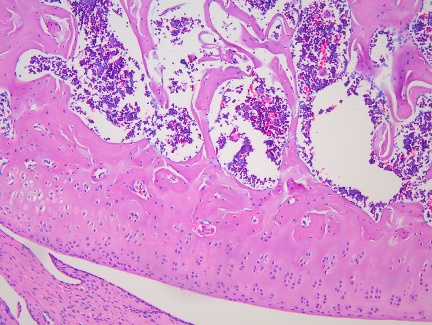


HE-A-blank-6 week


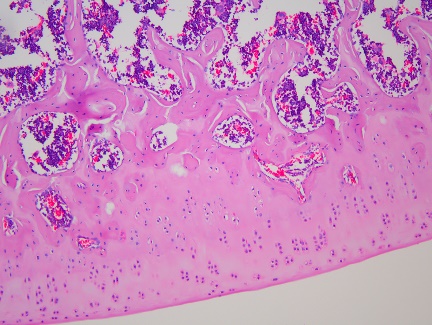


HE-A-blank-12 week


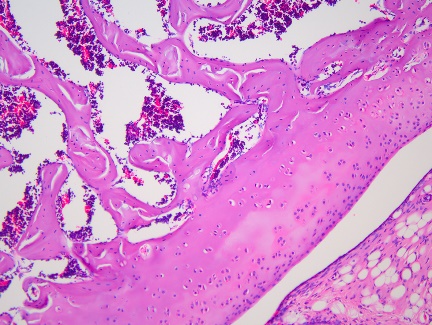


HE-A-SAMD3 protein-2 Week


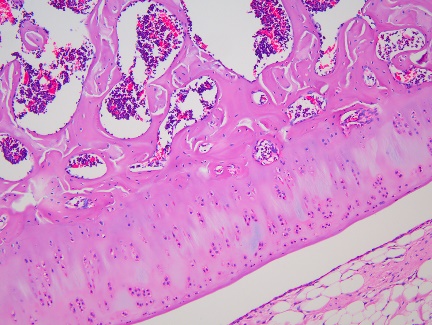


HE-A-SAMD3 protein-6 Week


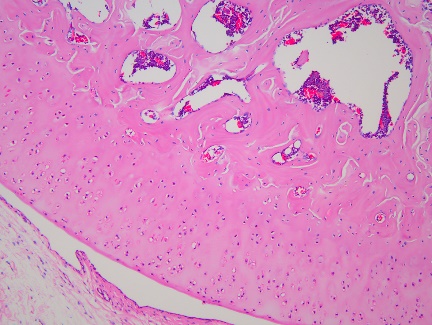


HE-A-SAMD3 protein-12 Week


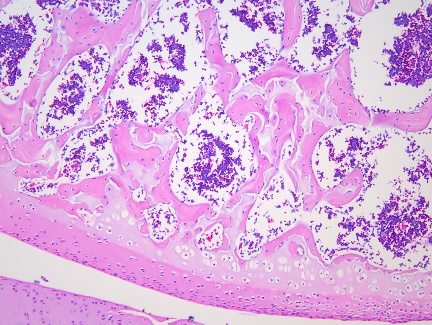


HE-A-SIS3-2 week


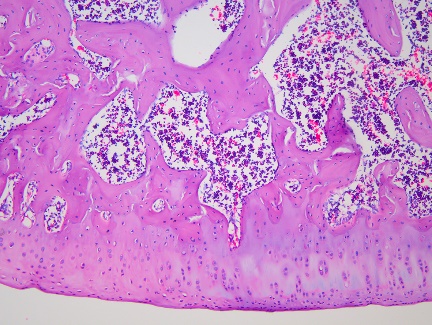


HE-A-SIS3-6 week


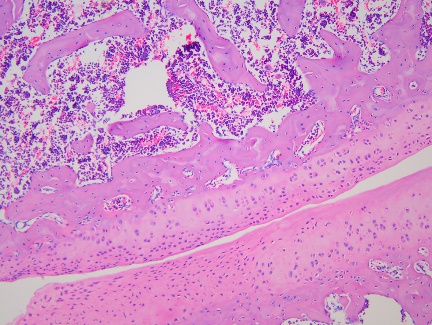


HE-A-SIS3-12 week


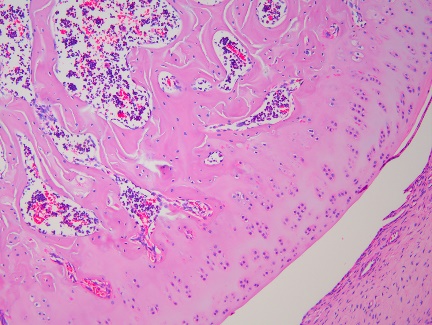


HE-B-ADAMTS-5 inhibitor-2 Week


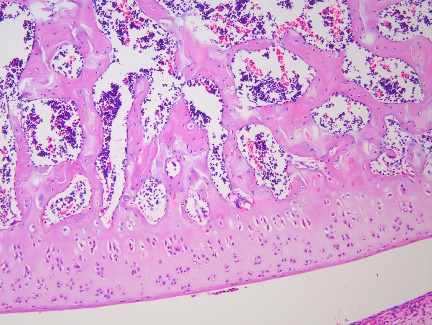


HE-B-ADAMTS-5 inhibitor-6 Week


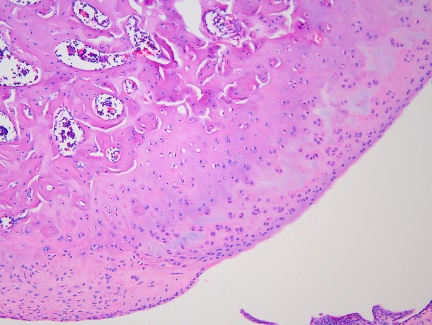


HE-B-ADAMTS-5 inhibitor-12 Week


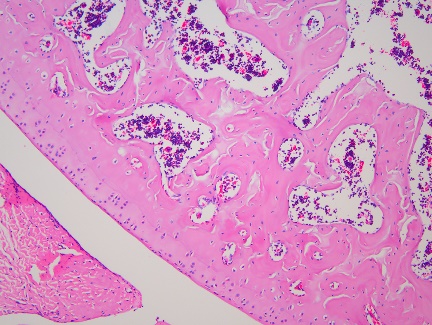


HE-B-blank-2 week


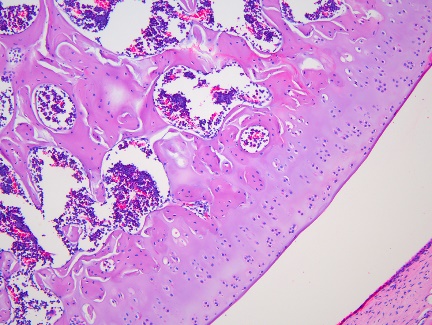


HE-B-blank-6 week


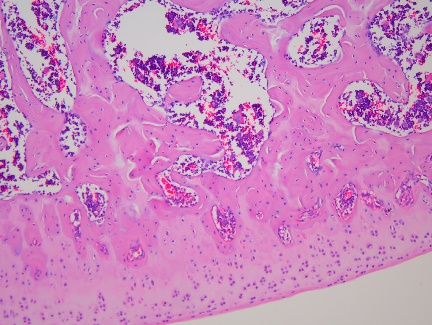


HE-B-blank-12 week


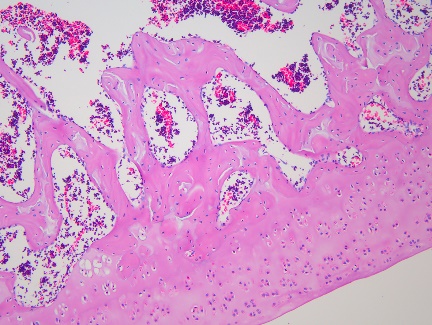


HE-B-miRNA-140 inhibitor-2 Week


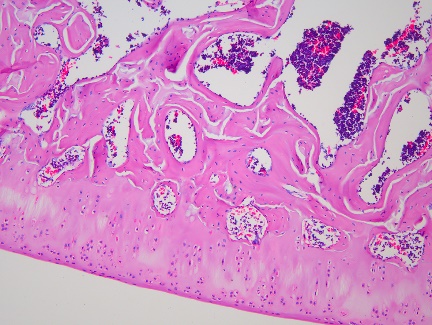


HE-B-miRNA-140 inhibitor-6 Week


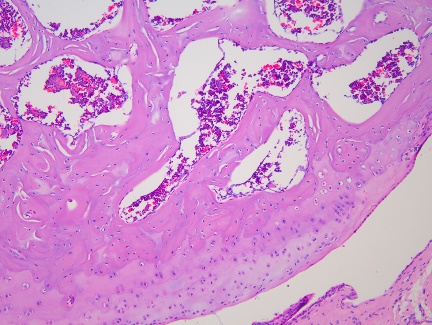


HE-B-miRNA-140 inhibitor-12 Week


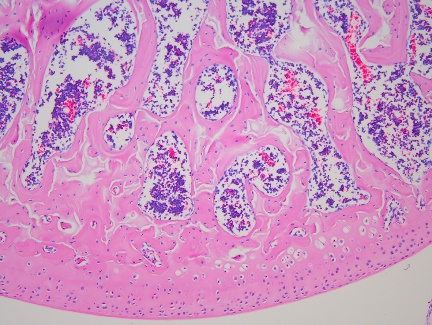


HE-B-miRNA-140 mimics-2 Week


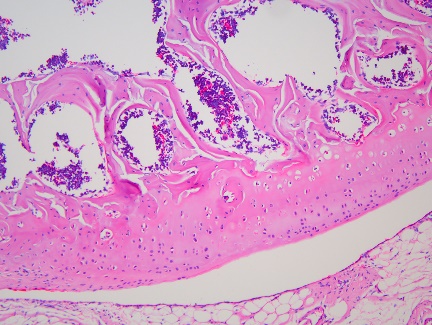


HE-B-miRNA-140 mimics-6 Week


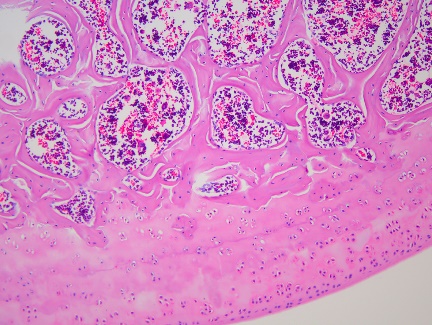


HE-B-miRNA-140 mimics-12 Week


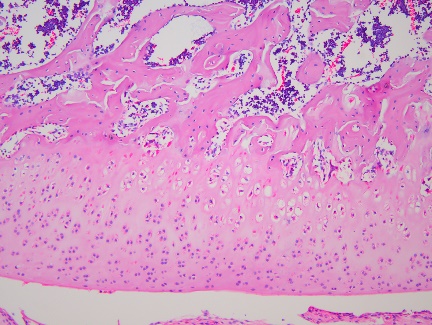


HE-B-Random RNA-2 week


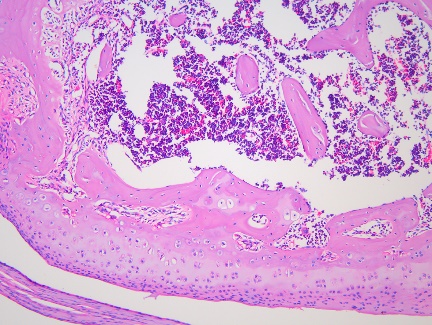


HE-B-Random RNA-6 week


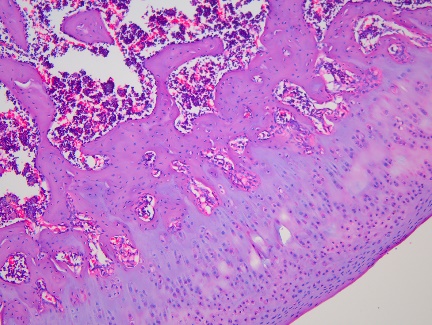


HE-B-Random RNA-12 week

# IHC


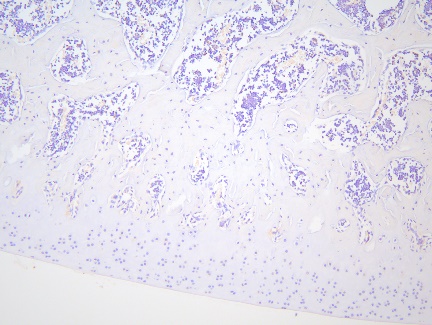


IHC-A-ADAMTS-5 inhibitor-2 week


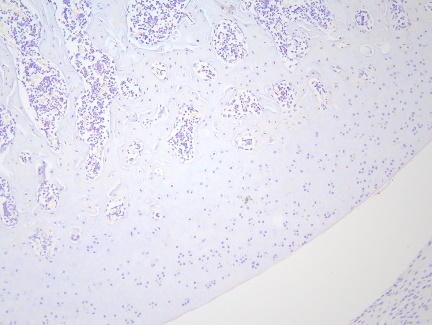


IHC-A-ADAMTS-5 inhibitor-6 week


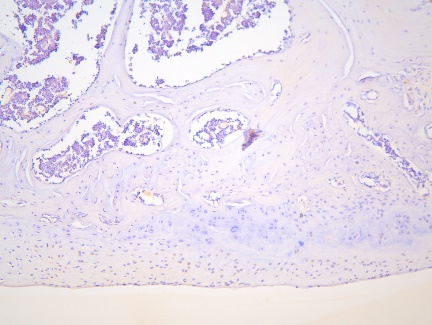


IHC-A-ADAMTS-5 inhibitor-12 week


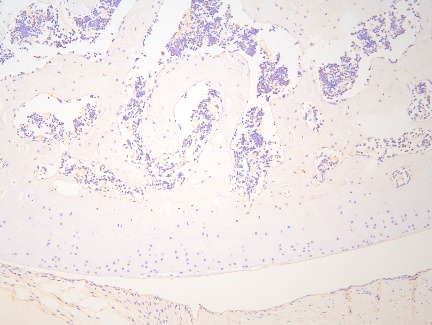


IHC-A-blank-2 week


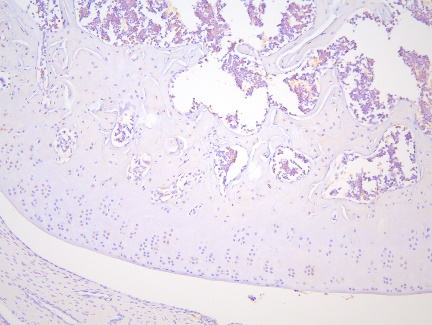


IHC-A-blank-6 week


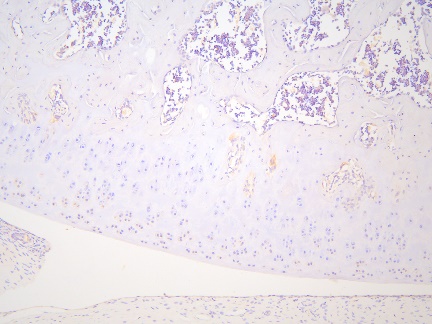


IHC-A-blank-12 week


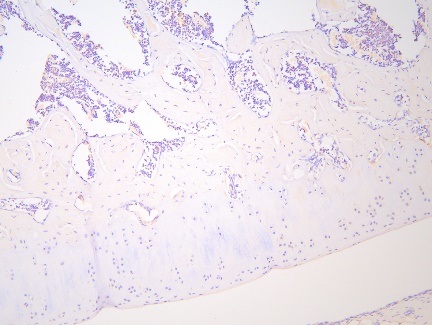


IHC-A-SIS3-2 week


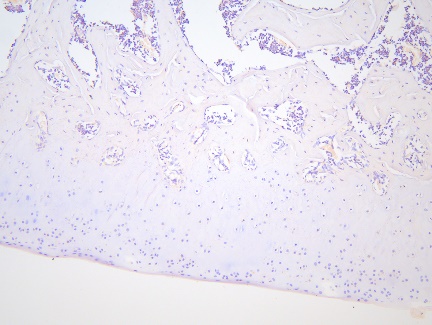


IHC-A-SIS3-6 week


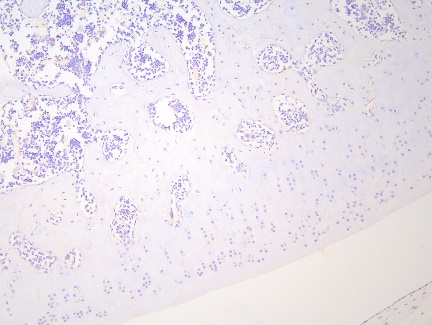


IHC-A-SIS3-12 week


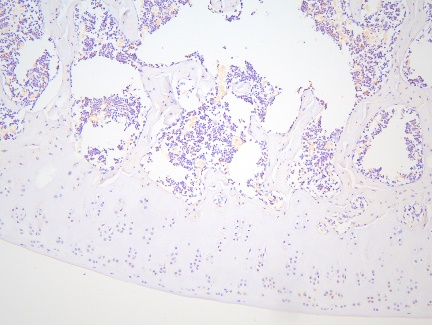


IHC-A-SMAD3 protein-2 week


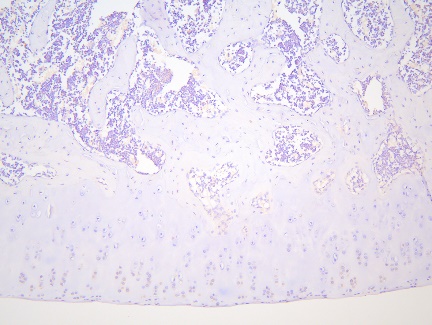


IHC-A-SMAD3 protein-6 week


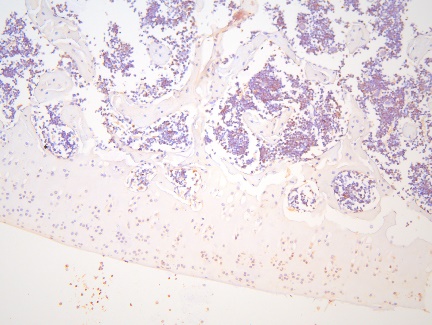


IHC-A-SMAD3 protein-12 week


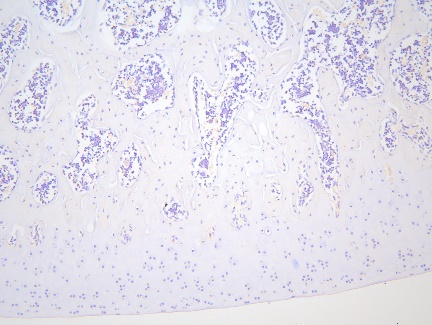


IHC-B-ADAMTS-5 inhibitor-2 week


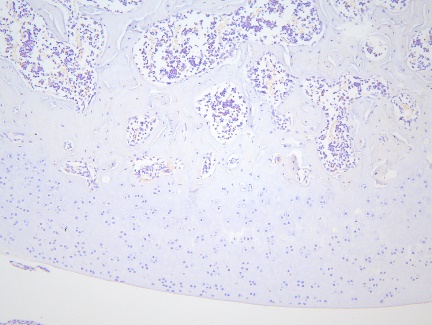


IHC-B-ADAMTS-5 inhibitor-6 week


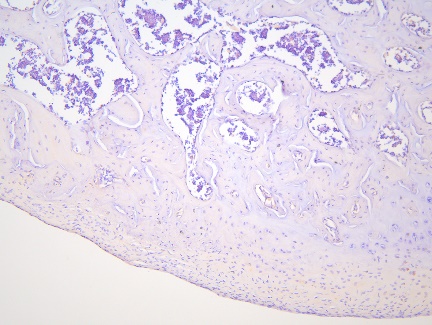


IHC-B-ADAMTS-5 inhibitor-12 week


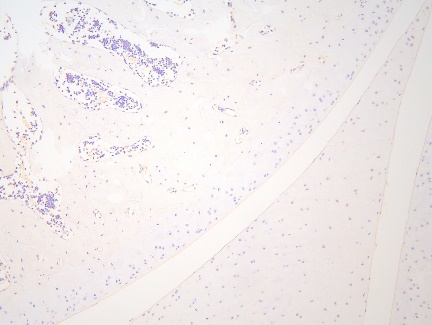


IHC-B-blank-2 week


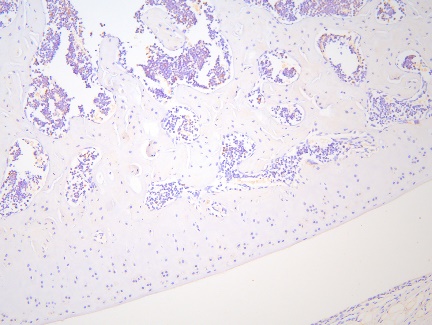


IHC-B-blank-6 week


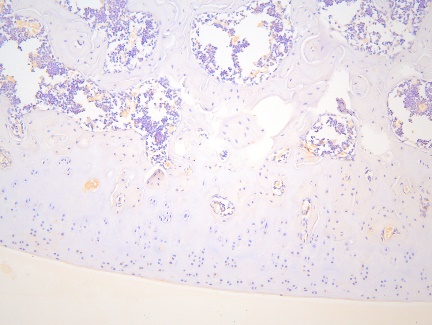


IHC-B-blank-12 week


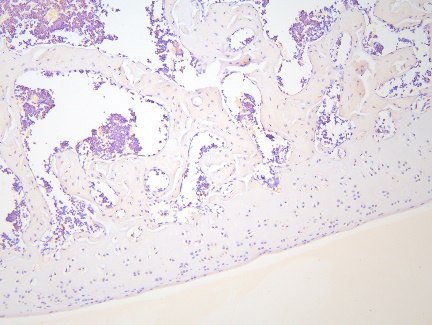


IHC-B-miRNA-140 inhibitor-2 week


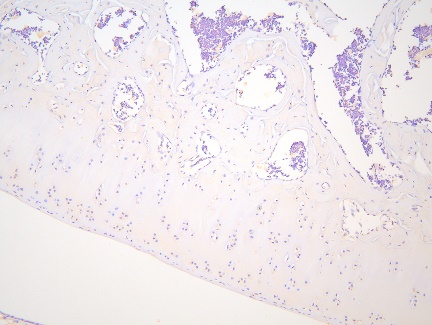


IHC-B-miRNA-140 inhibitor-6 week


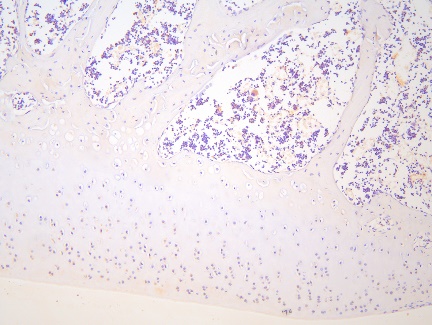


IHC-B-miRNA-140 inhibitor-12 week


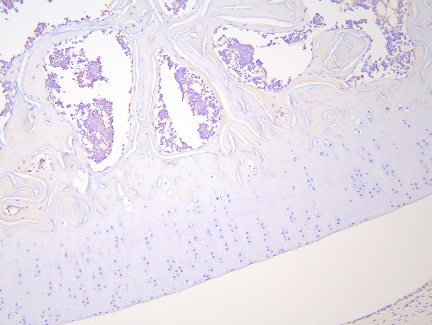


IHC-B-miRNA-140 mimics-2 week


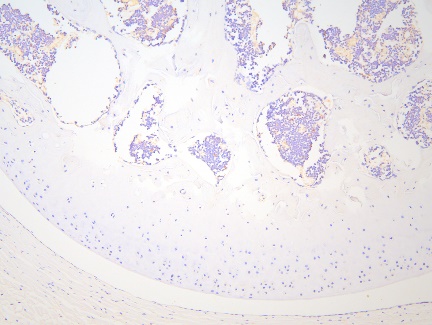


IHC-B-miRNA-140 mimics-6 week


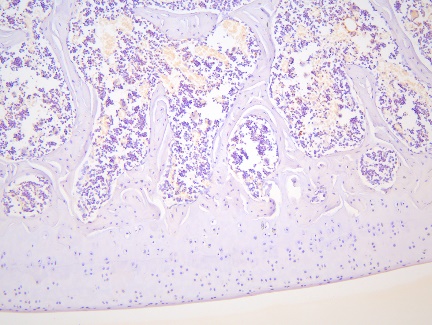


IHC-B-miRNA-140 mimics-12 week


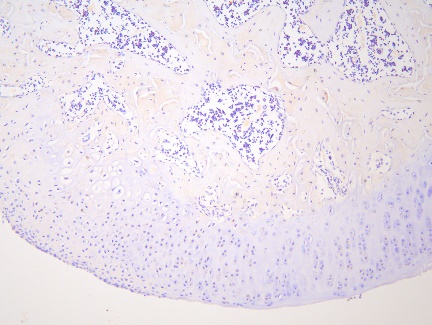


IHC-B-Random RNA-2 week


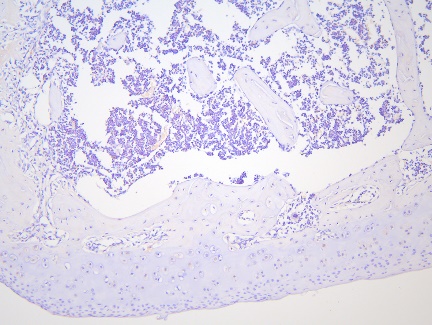


IHC-B-Random RNA-6 week


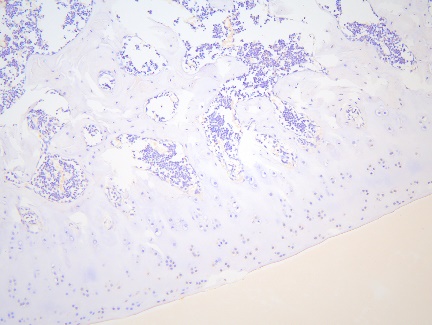


IHC-B-Random RNA-12 week


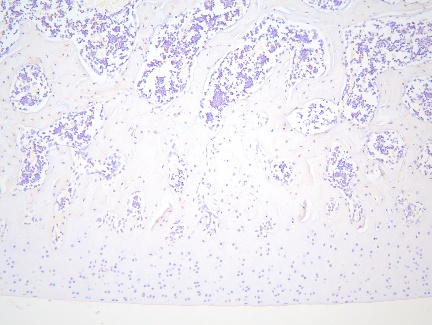


IHC-C-ADAMTS-5 inhibitor-2 week


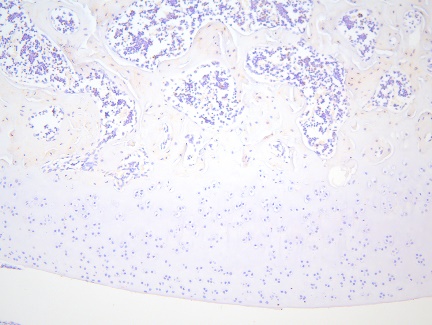


IHC-C-ADAMTS-5 inhibitor-6 week


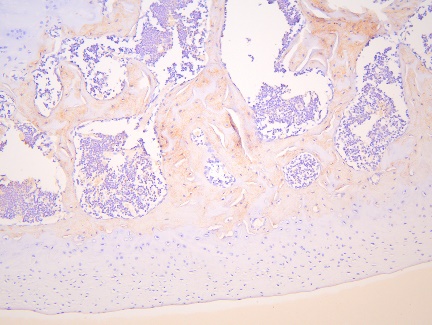


IHC-C-ADAMTS-5 inhibitor-12 week


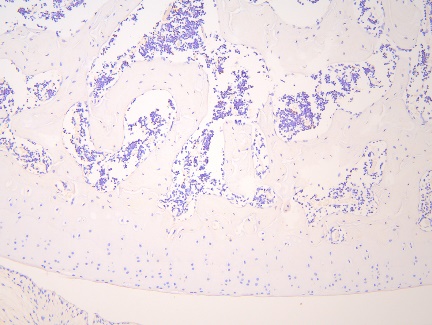


IHC-C-blank-2 week


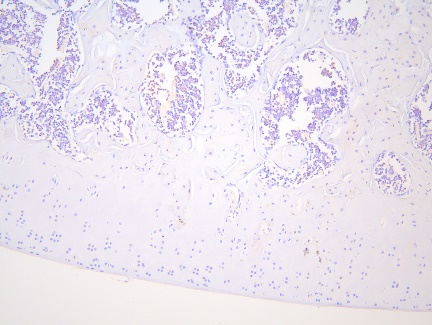


IHC-C-blank-6 week


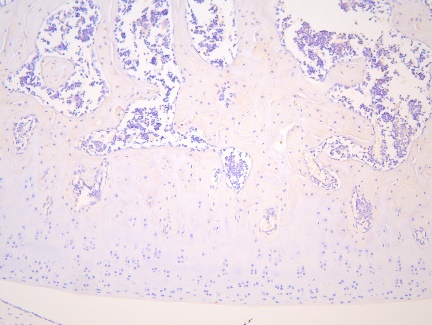


IHC-C-blank-12 week


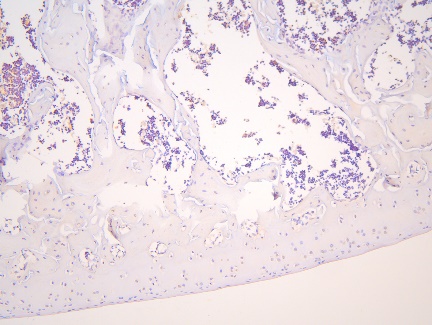


IHC-C-miRNA-140 inhibitor-2 week


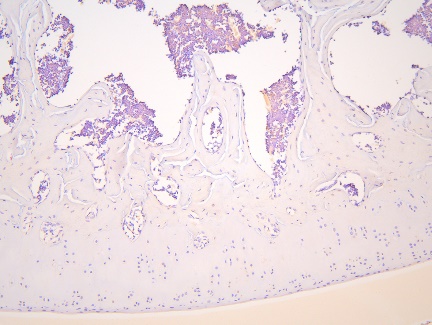


IHC-C-miRNA-140 inhibitor-6 week


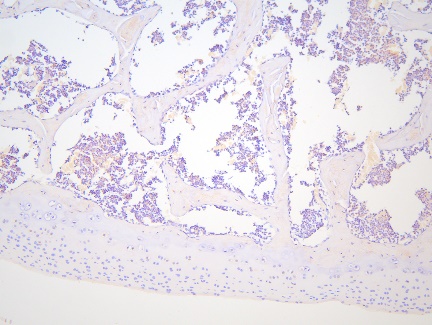


IHC-C-miRNA-140 inhibitor-12 week


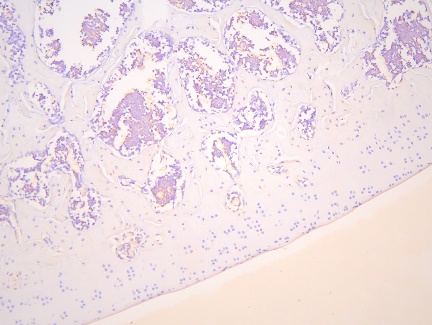


IHC-C-miRNA-140 mimics-2 week


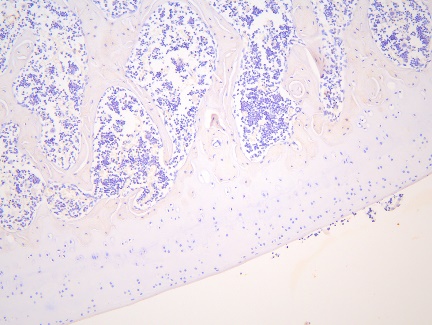


IHC-C-miRNA-140 mimics-6 week


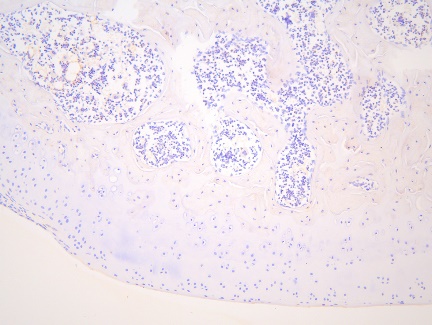


IHC-C-miRNA-140 mimics-12 week


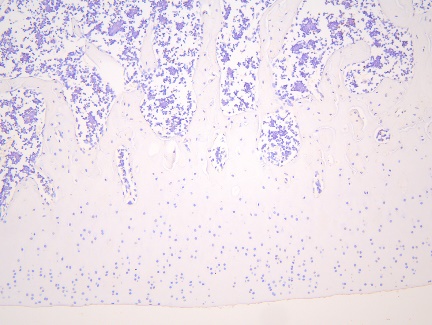


IHC-C-Random RNA-2 week


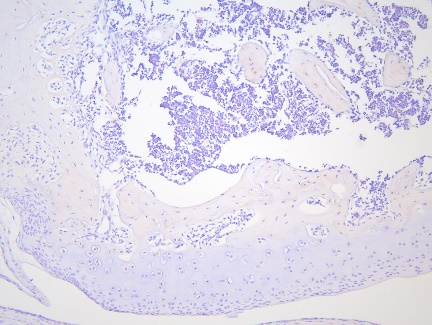


IHC-C-Random RNA-6 week


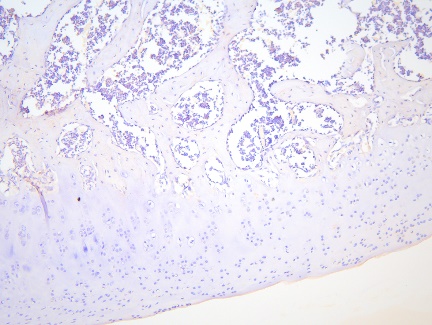


IHC-C-Random RNA-12 week


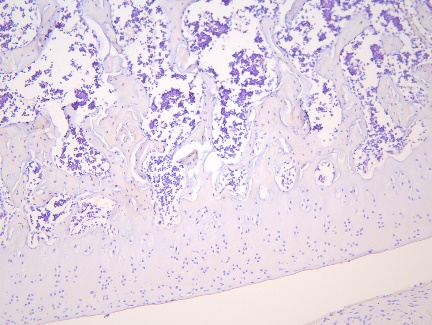


IHC-D-ADAMTS-5 inhibitor-2 week


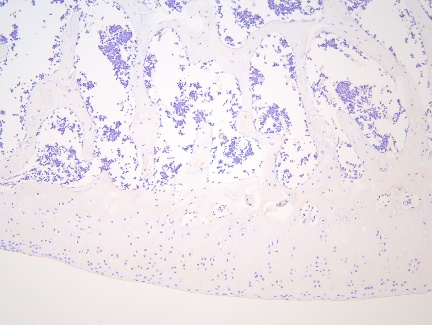


IHC-D-ADAMTS-5 inhibitor-6 week


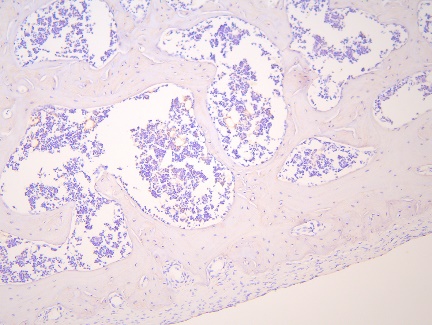


IHC-D-ADAMTS-5 inhibitor-12 week


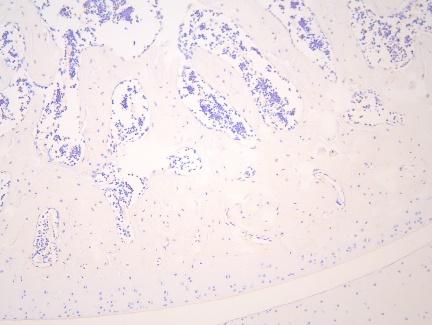


IHC-D-blank-2 week


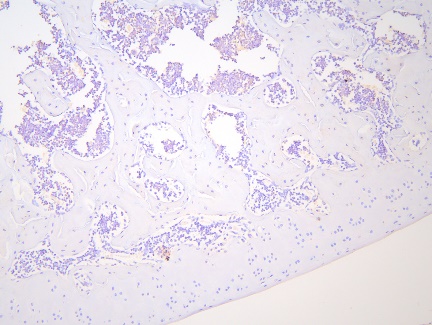


IHC-D-blank-6 week


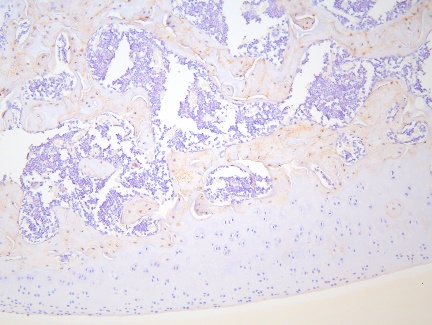


IHC-D-blank-12 week


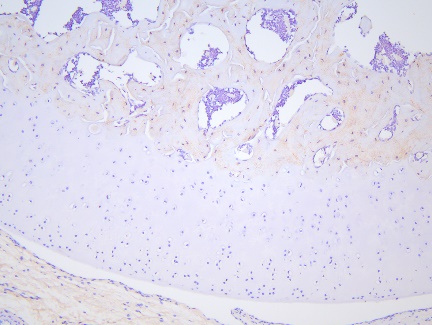


IHC-D-SIS3-2 week


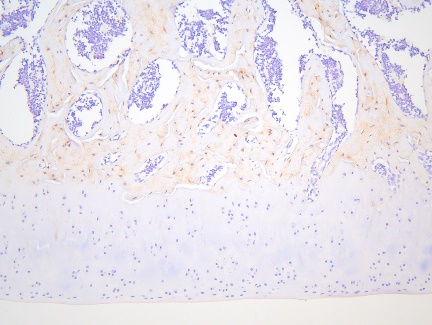


IHC-D-SIS3-6 week


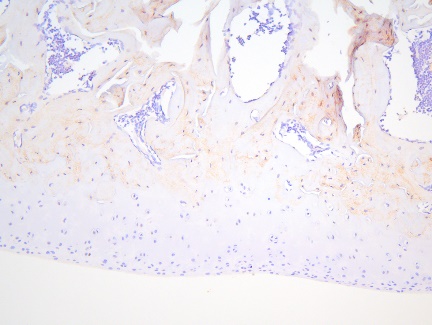


IHC-D-SIS3-12 week


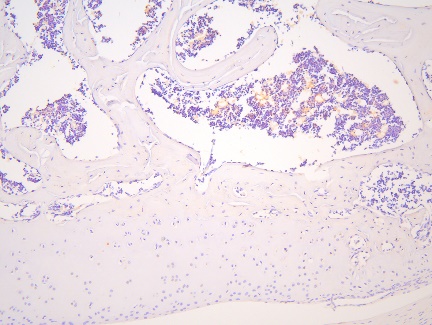


IHC-D-SMAD3 protein-2 week


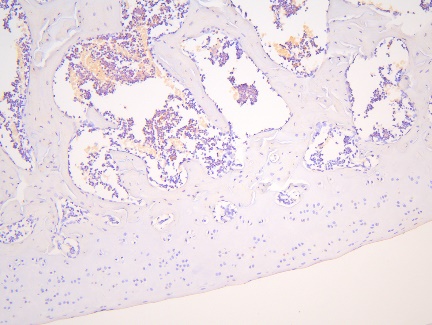


IHC-D-SMAD3 protein-6 week


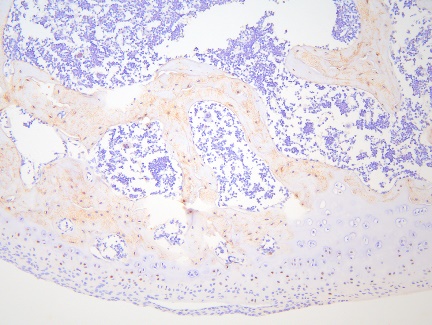


IHC-D-SMAD3 protein-12 week

# Safranin O stain


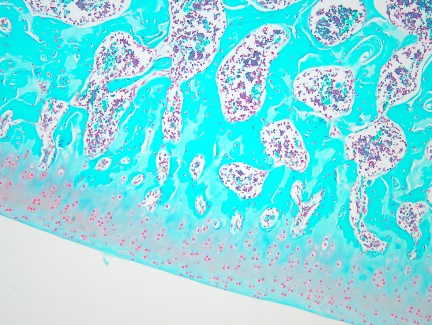


Safranin O stain-A-ADAMTS-5 inhibitor-2 week


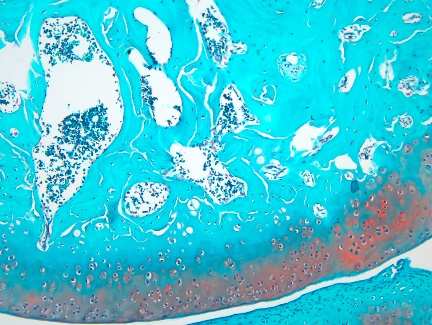


Safranin O stain-A-ADAMTS-5 inhibitor-6 week


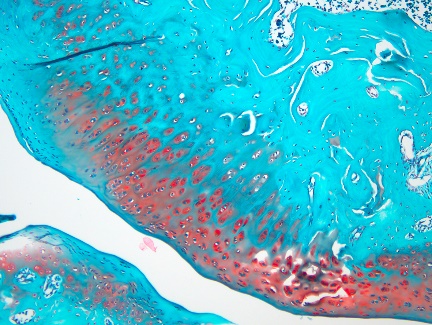


Safranin O stain-A-ADAMTS-5 inhibitor-12 week


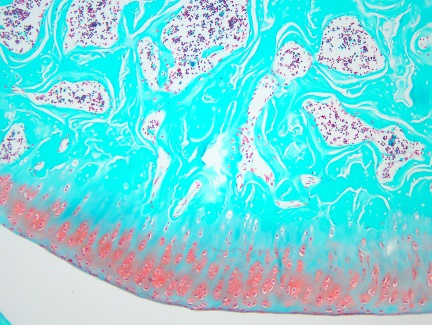


Safranin O stain-A-blank-2 week


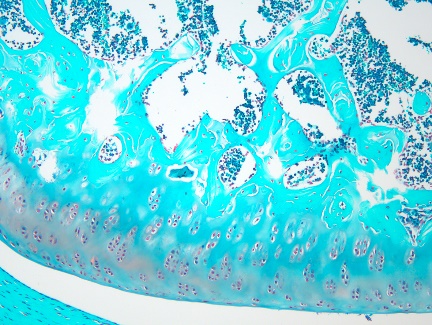


Safranin O stain-A-blank-6 week


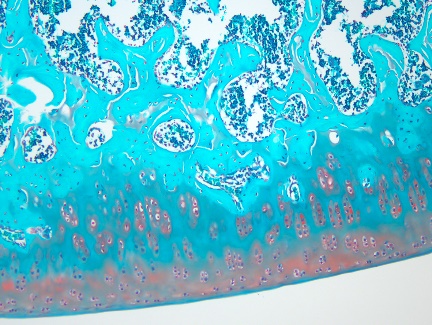


Safranin O stain-A-blank-12 week


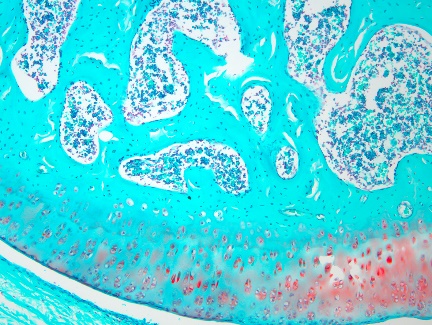


Safranin O stain-A-SIS3-2 week


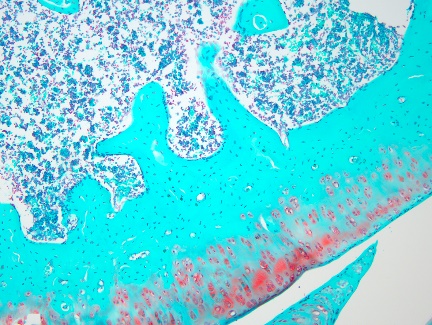


Safranin O stain-A-SIS3-6 week


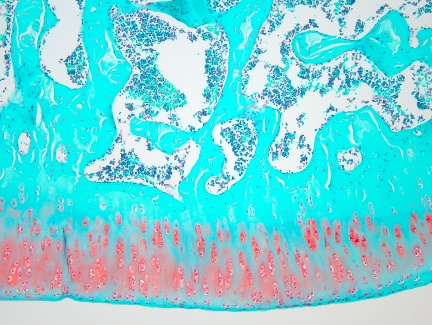


Safranin O stain-A-SIS3-12 week

Safranin O stain-A-SMAD3 protein-2 week

Safranin O stain-A-SMAD3 protein-6 week

Safranin O stain-A-SMAD3 protein-12 week

Safranin O stain-B-ADAMTS-5 inhibitor-2 week

Safranin O stain-B-ADAMTS-5 inhibitor-6 week

Safranin O stain-B-ADAMTS-5 inhibitor-12 week

Safranin O stain-B-blank-2 week

Safranin O stain-B-blank-6 week

Safranin O stain-B-blank-12 week

Safranin O stain-B-miRNA-140 inhibitor-2 week

Safranin O stain-B-miRNA-140 inhibitor-6 week

Safranin O stain-B-miRNA-140 inhibitor-12 week

Safranin O stain-B-miRNA-140 mimics-2 week

Safranin O stain-B-miRNA-140 mimics-6 week

Safranin O stain-B-miRNA-140 mimics-12 week

Safranin O stain-B-Random RNA-2 week

Safranin O stain-B-Random RNA-6 week

Safranin O stain-B-Random RNA-12 week
