# Supplementary figures and images for: Inhibition of SMAD3 effectively reduces ADAMTS-5 expression in the early stages of osteoarthritis
Source: BMC Musculoskelet Disord. 2023 Feb 17;24:130. doi: 10.1186/s12891-022-05949-8 (PMC9936734; doi:10.1186/s12891-022-05949-8)

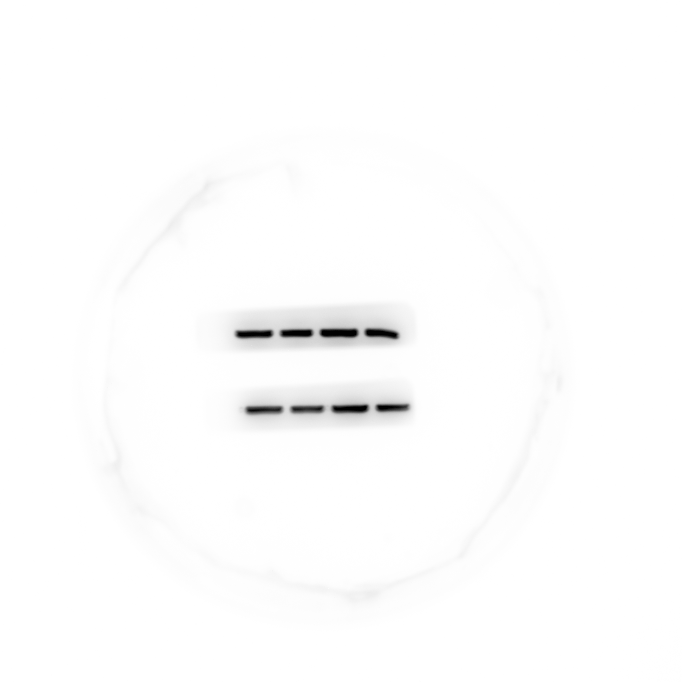

Supplement: Supplementary file 1 — Additional file 1. [file 12891_2022_5949_MOESM1_ESM.zip › cell sample-WB-ß-actin (blank-ADAMTS-5 inhibitor-SMAD3 protein-SIS3).docx]

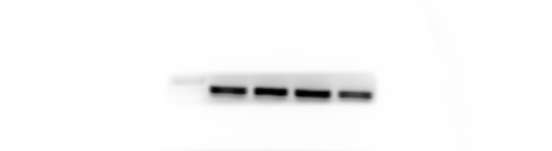

Supplement: Supplementary file 1 — Additional file 1. [file 12891_2022_5949_MOESM1_ESM.zip › cell sample-WB-ADAMTS-5(blank-ADAMTS-5 inhibitor-SMAD3 protein-SIS3).docx]

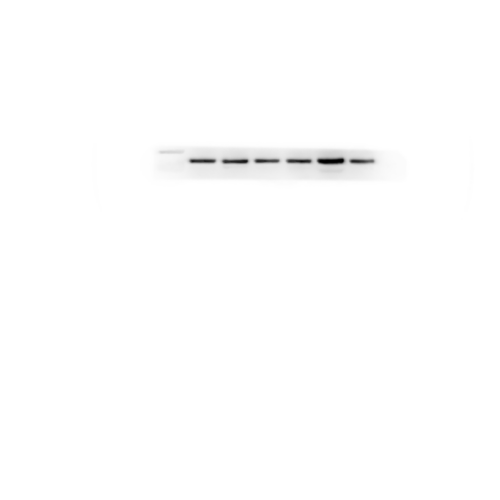

Supplement: Supplementary file 1 — Additional file 1. [file 12891_2022_5949_MOESM1_ESM.zip › cell sample-WB-ADAMTS-5(miRNA-140).docx]

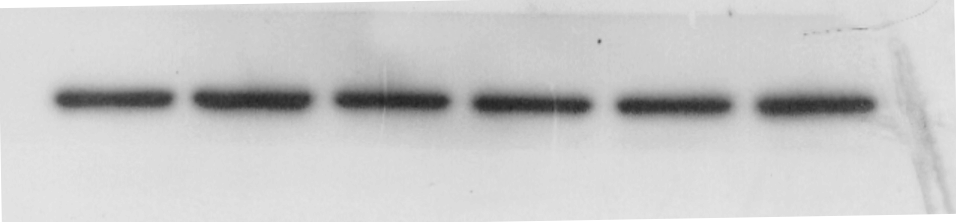

Supplement: Supplementary file 1 — Additional file 1. [file 12891_2022_5949_MOESM1_ESM.zip › cell sample-WB-ß-actin (miRNA-140).docx]

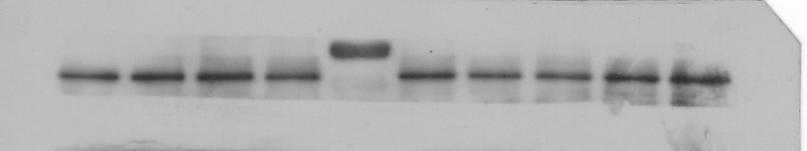

Supplement: Supplementary file 1 — Additional file 1. [file 12891_2022_5949_MOESM1_ESM.zip › tissue sample-WB-ß-actin-2 week(left_SIS3,right_miRNA-140).docx]

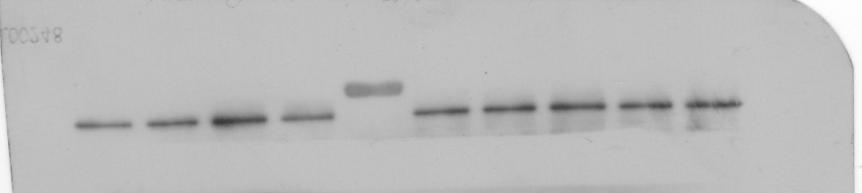

Supplement: Supplementary file 1 — Additional file 1. [file 12891_2022_5949_MOESM1_ESM.zip › tissue sample-WB-ß-actin-6 week(left_SIS3,right_miRNA-140).docx]

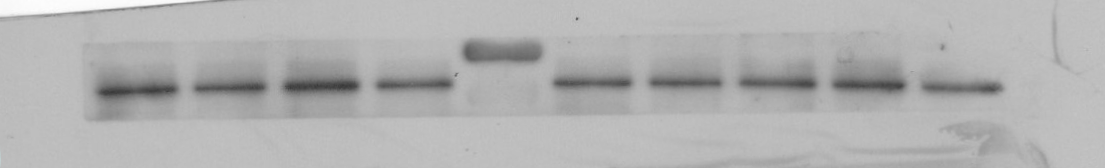

Supplement: Supplementary file 1 — Additional file 1. [file 12891_2022_5949_MOESM1_ESM.zip › tissue sample-WB-ADAMTS-5-12 week(left_SIS3,right_miRNA-140).docx]

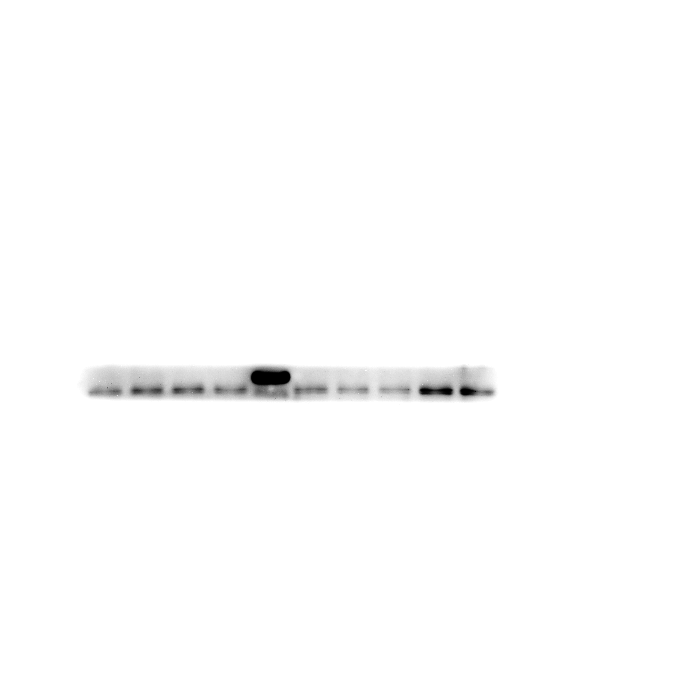

Supplement: Supplementary file 1 — Additional file 1. [file 12891_2022_5949_MOESM1_ESM.zip › tissue sample-WB-ADAMTS-5-2 week(left_SIS3,right_miRNA-140).docx]

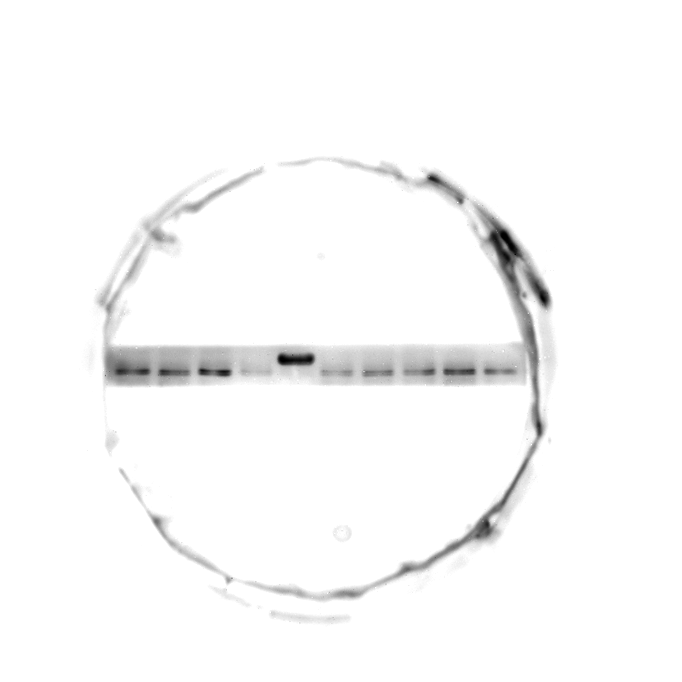

Supplement: Supplementary file 1 — Additional file 1. [file 12891_2022_5949_MOESM1_ESM.zip › tissue sample-WB-ADAMTS-5-6 week(left_SIS3,right_miRNA-140).docx]

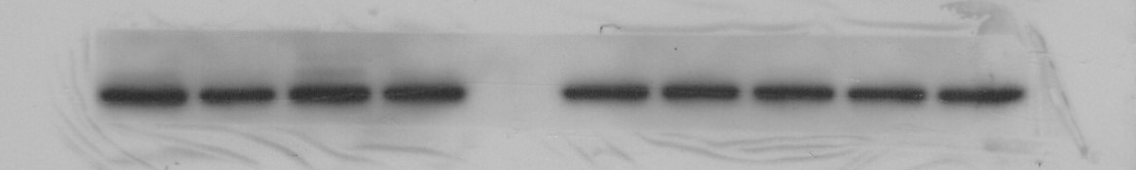

Supplement: Supplementary file 1 — Additional file 1. [file 12891_2022_5949_MOESM1_ESM.zip › tissue sample-WB-ß-actin-12 week(left_SIS3,right_miRNA-140).docx]

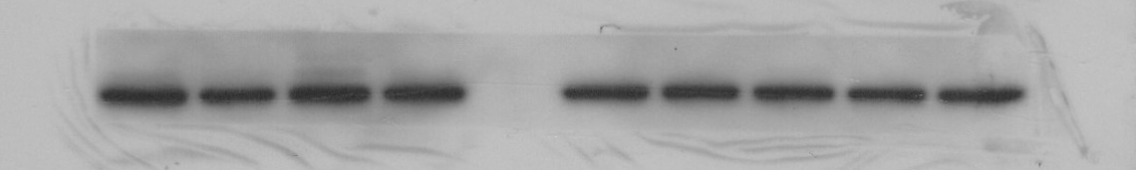

Supplement: Supplementary file 1 — Additional file 1. [file 12891_2022_5949_MOESM1_ESM.zip › tissue sample-WB-ß-actin-12 week(left_SIS3,right_miRNA-140).png]
